# Extracting epilepsy‐related information from unstructured clinic letters using large language models

**DOI:** 10.1111/epi.18475

**Published:** 2025-07-10

**Authors:** Shichao Fang, Ben Holgate, Anthony Shek, Joel S. Winston, Matthew McWilliam, Pedro F. Viana, James T. Teo, Mark P. Richardson

**Affiliations:** ^1^ Department of Basic & Clinical Neuroscience King's College London London UK; ^2^ King's College Hospital NHS Foundation Trust London UK; ^3^ Guy's and St Thomas' NHS Foundation Trust London UK

**Keywords:** electronic health records, epilepsy, large language models, natural language processing

## Abstract

**Objective:**

The emergence of large language models (LLMs) and the increasing prevalence of electronic health records (EHRs) present significant opportunities for advancing health care research and practice. However, research that compares and applies LLMs to extract key epilepsy‐related information from unstructured medical free text is under‐explored. This study fills this gap by comparing and applying different open‐source LLMs and methods to extract epilepsy information from unstructured clinic letters, thereby optimizing EHRs as a resource for the benefit of epilepsy research. We also highlight some limitations of LLMs.

**Methods:**

Employing a dataset of 280 annotated clinic letters from King's College Hospital, we explored the efficacy of open‐source LLMs (Llama and Mistral series) for extracting key epilepsy‐related information, including epilepsy type, seizure type, current anti‐seizure medications (ASMs), and associated symptoms. The study used various extraction methods, including direct extraction, summarized extraction, and contextualized extraction, complemented by role‐prompting and few‐shot prompting techniques. Performance was evaluated against a gold standard dataset, and was also compared to advanced fine‐tuned models and human annotations.

**Results:**

Llama 2 13b (a 13‐billion‐parameter LLM developed by Meta) demonstrated superior extraction capabilities across tasks by consistently outperforming other LLMs (F1 = .80 in epilepsy‐type extraction, F1 = .76 in seizure‐type extraction, and F1 = .90 in current ASMs extraction). Here, F1 score is a balanced metric indicating the model's accuracy in correctly identifying relevant information without excessive false positives. The study highlights the direct extraction showing consistent high performance. Comparative analysis showed that LLMs outperformed current approaches like MedCAT (Medical Concept Annotation Tool) in extracting epilepsy‐related information (.2 higher in F1).

**Significance:**

The results affirm the potential of LLMs in medical information extraction relating to epilepsy, offering insights into leveraging these models for detailed and accurate data extraction from unstructured texts. The study underscores the importance of method selection in optimizing extraction performance and suggests a promising avenue for enhancing medical research and patient care through advanced natural language processing technologies.

**Key points**

- Five popular open‐source large language models (LLMs) and six different extraction methods were compared.
- Llama 2 13b performed best in almost all aspects of information extraction.
- LLMs outperformed current existing approaches such as MedCAT (Medical Concept Annotation Tool).
- Results reveal some limitations of LLMs compared to human annotations.
- Applying LLMs to extract epilepsy‐related information from unstructured text is beneficial for epilepsy research.

## 1 INTRODUCTION

Epilepsy is characterized by repeated seizures due to abnormal neuronal activity.^1^ This abnormal activity temporarily affects multiple aspects of brain function, thereby significantly impacting the quality of life of the people affected.^2^ Treatment primarily involves medications, with ~70% of people with epilepsy successfully managing their seizures through this approach.^3^ The selection of these anti‐seizure medications (ASMs) is based on factors such as seizure type, age, gender, possible side effects, and general health. Therefore, it is essential to choose the right medication for each individual's unique situation. Moreover, for some people with epilepsy, even after trying multiple medications, their seizures remain difficult to control, a condition known as refractory epilepsy.^4^ Therefore, continued research into treatment options for epilepsy, particularly for refractory epilepsy, remains a significant priority in the field.^5^

The advent of the big data era and the widespread implementation of electronic health records (EHRs) have opened new opportunities for epilepsy research.^6^, ^7^ EHRs contain a wealth of patient health information, including disease history, treatments, and clinical outcomes. Researchers can use these data to analyze patterns of epileptic seizures, etiologies, and patient responses to different treatment regimens, with the potential to identify novel and effective treatment methods from large‐scale datasets.^8^

However, the use of EHRs also faces challenges.^9^, ^10^ Primarily, EHRs typically involve sensitive personal information, such as names, addresses, contact details, medical histories, laboratory and test results, and lifestyle information, making data protection a crucial concern. In addition, the degree of data structuring in EHRs varies. Demographics are generally stored in a structured format, whereas detailed information about the disease, such as disease history, treatment responses, and clinical outcomes, often appears in unstructured text forms, such as clinic letters.^11^ These unstructured clinic letters often exhibit a high degree of variability in expression, including diverse terminology, colloquial language, and spelling errors or inconsistencies in notation. This complexity significantly challenges the extraction and analysis of epilepsy‐related information, including epilepsy types, seizure types, current ASMs, and associated symptoms. Therefore, extracting epilepsy‐related information from unstructured clinic letters is a vital and pressing task in current epilepsy research. Successfully achieving this could significantly promote personalized treatment approaches for epilepsy, enhance the success rate of treatments, and pave new avenues for research in epilepsy.

In recent years, the rapid advancement of natural language processing (NLP) technologies has greatly facilitated the extraction of information from unstructured text in the medical field. Some existing studies have employed rule‐based methods to extract information from EHRs.^12^, ^13^, ^14^, ^15^ However, these methods are typically more effective for structured or semi‐structured data and face significant challenges when applied to unstructured text. In addition, rules‐based approaches lack generalizability; that is, rules developed for data from one hospital may not be applicable to another. Machine learning (ML) and deep learning models have shown promising results in extracting information from unstructured EHRs.^16^, ^17^, ^18^, ^19^, ^20^, ^21^, ^22^, ^23^ However, these models often require substantial time and resources for training for a specific domain. The effectiveness of the extraction is closely related to the training domain, and these models often lack direct generalization; instead, they require fine‐tuning. Although some studies utilize administrative health care data, such as International Classification of Diseases (ICD) codes, to identify epilepsy and its subtypes, these methods first require the deployment and maintenance of ICD coding systems within hospitals.^24^, ^25^, ^26^, ^27^ Moreover, the performance of these methods is significantly affected by the ICD coding version and granularity, as mapping between different ICD versions is challenging and often involves merging or splitting codes, which limits the generalizability of such methods.^28^, ^29^ Furthermore, it is difficult to balance sensitivity and specificity in these approaches.^24^, ^25^, ^26^, ^27^ In contrast, the use of NLP techniques to extract information directly from clinic letters offers greater flexibility in data format and enables the extraction of a wider range of informational elements. This approach also provides an important avenue for effectively deriving valuable insights when only unstructured data are available.

In contrast to smaller ML models, due to pre‐training on vast amounts of data, large language models (LLMs) often exhibit higher generalization capabilities and can be applied to different domains without the need for additional fine‐tuning. LLMs, such as the Generative Pre‐trained Transformer (GPT) and Pathways Language Model (PaLM) series, present significant opportunities in the medical field.^30^ GPT4 and PaLM 2 have demonstrated capabilities close to that of human clinical experts by achieving notable results on U.S. Medical Licensing Examinations.^31^, ^32^, ^33^, ^34^ In terms of prediction and prognosis, Foresight has demonstrated considerable efficacy.^35^ Our previous study has shown that Llama 2 can effectively extract a patient's seizure frequency at a given point in time from unstructured, free‐text EHRs.^36^ Although studies have examined using LLMs to extract unstructured medical information for seizure outcomes or Parkinson's disease,^37^, ^38^ the published research that compares and applies LLMs to extract several key epilepsy‐related information from unstructured medical free text remains limited.^39^

In this study, we explore a novel approach of using LLMs, including Llama 3,^40^ an opensource LLM released by Meta in April 2024, Llama 2^41^ (Meta, released in July 2023), and Mistral^42^ (Mistral AI, September 2023), to extract epilepsy‐related information from unstructured clinic letters in real‐world hospitals. Specifically, we explore whether these LLMs can accurately identify and extract key information about a patient's epilepsy types, seizure types, current ASMs, and the associated symptoms. The primary contribution of this research lies in introducing new methods and technologies to the field of medical information extraction, particularly in epilepsy research. This study explores the comparison and application of various LLMs and extraction methods in the field of epilepsy. In addition, it reveals some limitations of LLMs in this area by analyzing the discrepancies with human annotations. The method proposed in this study demonstrates the potential of LLMs to utilize large patient databases in research settings. By extracting more precise clinical information from large‐scale patient databases, this approach may support downstream tasks in the future, thereby significantly enhancing personalized treatment strategies for epilepsy.

## 2 METHODS

### 2.1 Ethical approval

This project operated under UK Health Research Authority (HRA) London South East Research Ethics Committee approval (reference 18/LO/2048 and renewed 24/LO/0057) granted to the King's Electronic Records Research Interface (KERRI) with data research opt‐out. This study was approved by the KERRI committee at King's College Hospital (KCH) for purposes of evaluating NLP for Epilepsy (approved February 2021) with local institutional oversight.

### 2.2 Data source

In this study, we accessed 25 401 EHRs including demographics and clinic letters from KCH in London, spanning from January 1, 2013 to December 31, 2019. We included only EHRs of patients with epilepsy 18 years of age or older to avoid the biases and complexities introduced by pediatric patients. KCH's internal data management platform made it straightforward to filter the required data.

We confined analysis to EHRs generated during attendances in secondary or tertiary care epilepsy ambulatory clinics or outpatient clinics. In the UK, these EHRs typically consist of an electronic note generated by the clinician (typically a neurologist or epilepsy nurse specialist), which are sent in the form of a letter to the patient and to the primary care physician involved in the patient's care. The letter usually includes all relevant aspects of the patient's history, examination, investigations, treatment, progress, and management plans. There may also be additional notes related to these clinic attendances, such as communications with other health care professionals, the patient, or their professional carers. Collectively, we refer to these EHRs as “clinic letters.”

To ensure the representativeness of our research sample, we randomly selected 280 clinic letters from the filtered dataset for our study. We chose 280 cases because, on the one hand, it provides a feasible workload for human annotation to establish a gold standard annotation; on the other hand, this sample size aligns with the data volumes used in similar studies.^43^, ^44^ Each case belonged to an individual visit of an individual patient. This random sampling method helps minimize sample selection bias and ensures the broad applicability of our research findings.

Our dataset recognizes that patients may experience multiple types of seizures; for example, a patient may have both generalized and focal seizures. This multifaceted approach to categorizing seizures—into generalized, focal, and unknown—provides a more comprehensive understanding of the patient's condition, essential for tailoring treatment and management strategies. The category ‘unknown’, in relation to epilepsy type or seizure type, means the clinic letter mentions that the patient has epilepsy and/or seizures but the letter does not provide the specific epilepsy type or seizure type.

We concentrated on the ASMs that patients were actively taking at the time of their clinic visits, rather than their historical or newly prescribed medications. This included medications that patients had been continuously taking from their last consultation to the current one. This focus not only presented a challenge in data extraction but also added significant value to our research. It allowed us to analyze the current medication trends and changes in prescriptions more effectively, offering insights into real‐world patient management.

The selection of the most common associated symptoms was driven by their prevalence as side effects of ASMs, based on expert selection in the previous study.^45^

### 2.3 Large language models

Given the sensitivity of real patient data in this study, protecting patient privacy is paramount and required by UK law. Therefore, any non‐open source LLMs that require internet connectivity are not considered.^46^ In addition, considering the computational costs (run time and video memory, which are affected by the model size) and the goal of making the technology accessible to a broader audience, we employed only relatively small‐scale LLMs. Therefore, we selected LLMs from the Llama and Mistral series. These open‐source models can be deployed locally, such as within a hospital's internal system. This choice provides a prerequisite for data security and privacy protection. Specifically, we experimented with Llama‐3‐8B‐Instruct, Llama‐2‐7b‐chat‐hf, Llama‐2‐13b‐chat‐hf, Mistral‐7B‐Instruct‐v0.1, and Mixtral‐8x7B‐v0.1 for comparison. These model names contain technical information about their configurations, which we clarify here to aid interpretation. For instance, “8B” or “7b” refer to the parameter size of the model (8 billion or 7 billion parameters, respectively). A higher parameter count generally enables more complex understanding but also requires greater computational resources. LLMs with these parameter sizes allow them to be implemented in most computational environments. Moreover, these versions of the models are at the forefront of performance among open‐source LLMs.^40^, ^41^, ^42^ This selection thus balances computational feasibility with state‐of‐the‐art capabilities, making these models particularly suitable for extracting detailed epilepsy‐related information from unstructured clinical texts.

### 2.4 Extraction

LLMs often interact through a question‐and‐answer format, where prompt engineering plays a crucial role. Prompting refers to the way in which requests or questions are posed to the LLM. These inquiries are known as prompts. The quality of prompting directly impacts the quality of the model's responses.^47^

LangChain is an open‐source NLP tool designed to facilitate developers in building and deploying applications related to language generation more easily. It introduces the concept of “chain,’ a sequence of multiple, layered processes where each chain contains different prompts, allowing for a step‐by‐step refinement in data extraction. This approach enables progressively more precise information retrieval by stacking and combining these chains. Each chain within the system focuses on a specific aspect of the prompt, contributing to a more detailed and fine‐tuned data processing workflow. This flexibility allows for customization and optimization to suit specific research needs.^48^

**FIGURE 1:**
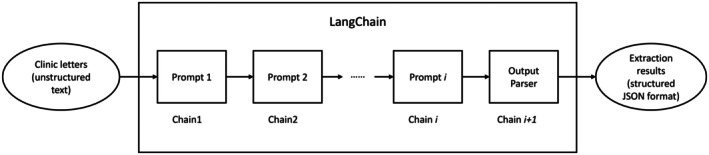
The overall extraction process. Unstructured clinic letters are sequentially processed through a series of prompt chains within the LangChain framework. Each chain (e.g., Chain 1 to Chain i) applies a specific prompt to transform or extract relevant information. The final output parser converts the processed data into structured JSON format. JSON, JavaScript Object Notation.

In this study, we integrated LangChain with different LLMs to create systems for extracting epilepsy‐related information from unstructured clinic letters. The overall extraction process is illustrated in Figure 1. An example has been shown in Appendix S1. For different categories of information, we utilized various prompts, enabling LLMs to generate responses based on our specific prompts. All experiments were conducted on a NVIDIA DGX system at KCH, equipped with 8 Tesla V100‐SXM2 Graphics Processing Units (GPUs) with a total of 256GB of video memory.

Our research focused on four key aspects of epilepsy‐related information: epilepsy type, seizure type, current ASMs, and associated symptoms. These were chosen for their clinical relevance and potential to yield insights into the multifaceted nature of the condition. Details of ASMs and associated symptoms are shown in Table 1.

**TABLE 1:** Patient characteristics.

Category	Subcategory	Count (percentage)
Gender	Female	142 (51.0%)
	Male	138 (49.0%)
Age group	21–30 years	73 (26.1%)
	31–40 years	68 (24.3%)
	41–50 years	57 (20.4%)
	51–60 years	42 (15.0%)
	18–20 years	25 (8.9%)
	61–70 years	12 (4.2%)
	71–80 years	3 (1.1%)
Epilepsy type	Unknown	143 (51.1%)
	Focal	81 (28.9%)
	Generalised	31 (11.1%)
	Combined generalised and focal	3 (1.1%)
Seizure type	Unknown seizure	144 (51.4%)
	Focal seizure	90 (32.1%)
	Generalised seizure	40 (14.2%)
Associated symptom	Depression	20 (7.1%)
	Headache	20 (7.1%)
	Lethargy	20 (7.1%)
	Anxiety	14 (5.0%)
	Dizziness	5 (1.8%)
	Nausea	2 (0.7%)
	Rash	1 (0.3%)
Current ASMs	Levetiracetam	63 (22.6%)
	Carbamazepine	61 (21.8%)
	Lamotrigine	59 (21.2%)
	Sodium volproate	53 (18.9%)
	Clobazam	38 (13.6%)
	Lacosamide	22 (7.9%)
	Topiramate	21 (7.5%)
	Zonisamide	17 (6.1%)
	Phenytoin	15 (5.4%)
	Gabapentin	7 (2.5%)
	Oxcarbazepine	7 (2.5%)
	Perampanel	6 (2.1%)
	Pregablin	6 (2.1%)
	Clonazepam	5 (1.7%)
	Brivaracetam	3 (1.1%)
	Ethosuximide	3 (1.1%)
	Phenobarbital	3 (1.1%)
	Rufinamide	3 (1.1%)
	Acetazolamide	2 (0.7%)
	Cannabidiol	2 (0.7%)
	Primidone	2 (0.7%)
	Retigabine	1 (0.3%)
	Vigabatrin	1 (0.3%)

Abbreviation: ASM, anti‐seizure medication.

#### 2.4.1 Extraction methods

In our study, we employed prompt engineering as a pivotal technique for extracting epilepsy‐related information from unstructured text using LLMs. Prompt engineering involves crafting specific prompts or questions to guide the LLMs in generating the desired output.^49^, ^50^

##### Direct extraction (DE)

This method involved inputting the text directly into the LLM and posing our query. The model processed the text and provided an answer based on the input data without additional summarization or contextualization.

##### Summarized‐direct extraction (SDE)

Initially, the LLM was prompted to summarize the text, focusing on aspects relevant to our query about the patient. This created a summarized version of the text, which was then used for DE. The summary served as a refined input, potentially allowing for more focused and accurate extraction of information.

##### Contextualized‐direct extraction (CDE)

Here, we first provided the LLM with relevant contextual information, such as different types of epilepsy and their subcategories.^51^ After establishing this context, we posed our question, enabling the model to generate responses that were more informed and precise.

##### Contextualized–summarized direct extraction (CSDE)

Similar to the previous method, we first gave the LLM relevant background knowledge. The model then created a summary incorporating this context, followed by the extraction process. This method combined the benefits of contextualization with the efficiency of summarization.

##### Role prompting extraction (RPE)

In this approach, we assigned a specific role to the LLM, such as a “Professional Medical Information Extractor” or an “Expert Epilepsy Neurologist.” Previous studies suggest that assigning a specific role to an LLM may enhance its performance in particular domains.^52^ LLMs have a built‐in role‐assignment mechanism, where simply adding “You are a [role]” to the template is typically sufficient to achieve the desired role effect. In this study, we selected a human‐like role (Expert Epilepsy Neurologist) and a machine‐like role (Professional Medical Information Extractor), both of which are suitable for information extraction tasks. This design allows us to systematically compare the effects of different role settings on extraction outcomes.

##### Few‐shot prompting extraction (FSPE)

Unlike zero‐shot prompting, where extraction was performed without any examples, few‐shot prompting involved providing the LLM with a small number of input–output examples. These examples served as a guide, helping the LLM to understand the format and context of the desired responses.^49^, ^52^

Each model utilized two principal extraction methods: DE and SDE. Due to the intricate nature of epilepsy types and seizure types, which encompass various subcategories, we incorporated two additional methods, CDE and CSDE, specifically for the epilepsy type and seizure type extraction. These methods were designed to enhance the granularity and accuracy of epilepsy type and seizure type information extraction by introducing more detailed background.

To explore whether RPE and FSPE could improve the extraction performance, we selected the model and methods with optimal results from these four extraction tasks and then allocated roles and provided few‐shot examples for comparison. In the case of RPE, we established two roles: “Professional Medical Information Extractor” and “Expert Epilepsy Neurologist.” For FSPE, we compared the effects with providing one example (one‐shot prompting) and four examples (few‐shot prompting). It is important to note that our examples included the entirety of the clinic letter. An excess of examples can cause the tokenizer in LLMs to generate too many tokens, leading to GPU memory overflow.

### 2.5 Gold standard annotation

To construct the gold standard dataset, we selected 280 clinic letters and defined the categories that required annotation. Each of these 280 clinic letters was independently reviewed by two epileptologists, both with post‐specialty residency training and substantial experience in epilepsy care. The first epileptologist has 24 years of clinical practice, whereas the second has 6 years, and their entire clinical practice is dedicated to patients with epilepsy. The epileptologists were tasked with marking the presence of each category mentioned in the letters with a “1,” and the absence with a “0.” Subsequently, we compared the annotations provided by the two experts. For instances where both experts agreed, their annotations were retained without modification. In cases where discrepancies arose, a consensus meeting was convened, involving both experts and our research team. During this meeting, a third annotation was conducted to resolve the differences and arrive at the final decision. In addition, we calculated the Cohen's kappa^53^ score for the annotations provided by the two epileptologists to demonstrate the inter‐annotator agreement. Through this approach, we established a high‐quality reference standard to assess and validate the accuracy of the epilepsy‐related information extracted by LLMs.

It is noteworthy that during the annotation process, annotators exclusively annotated information that was explicitly described in the clinic letters. This enables us to more effectively investigate the accuracy of LLMs in extracting information from the letters, rather than producing outcomes based on reasoning or speculation. For example, a clinic letter might describe that the patient missed today's appointment; thus no information can be extracted from this letter.

### 2.6 Evaluation

In this study, we evaluated our methods on four key extraction tasks: epilepsy type, seizure type, current ASMs, and associated symptoms. To conduct a thorough examination, we employed five different LLMs for information extraction from 280 clinic letters. The performance of each model was evaluated rigorously by comparing the extracted data against a gold standard dataset. This comparison enabled the calculation of recall, precision, and F1 scores, thereby providing a robust measure of each model's efficacy. As each extraction task involves multiple categories, it is necessary to compute the true positives (TPs), false positives (FPs), and false negatives (FNs) for all categories assigned to each data point. Because a single data point may involve multiple instances of TPs, FPs, and FNs across different categories, it is not feasible to calculate TPs, FPs, and FNs solely based on the 280 data points. Instead, we compute these values over all annotated label instances across the dataset, aggregating across the 280 data points and all defined categories. Accordingly, the overall precision, recall, and F1 score are calculated based on the total counts of TPs, FPs, and FNs from the entire dataset. To better quantify the uncertainty of our evaluation results due to the finite sample size, we report the standard error (SE) for each metric. These SEs are estimated using standard binomial approximation formulas: for precision and recall, the SE is computed as the square root of the metric multiplied by 1 minus the metric, divided by the corresponding sample size (TP + FP or TP + FN); for F1 score, the SE is approximated similarly, treating the F1 value as a proportion and using the total number of data points (280) as the sample size.

To mitigate potential uncertainties arising from model randomness, we set the temperature parameter to near‐zero (.00001). We conducted each extraction task four times for every model and method to observe variability, thereby providing a more comprehensive assessment of stability and consistency. In addition, we cleared the cache after running each case extraction to avoid model memory affecting the outcomes, thereby ensuring the reliability of our results. We averaged the results of the four extractions (F1 scores and time taken) to obtain the final values.

To evaluate the performance of different combinations of models and methods, we employed statistical comparisons based on F1 scores. For each type of extraction task, all possible pairs of model–method combinations were compared. Using the extraction results from 280 data samples, we calculated *p*‐values via paired *t* tests, a statistical method commonly used to assess whether the means of two paired groups differ significantly (*p* < 0.05).^54^ Given the limited size of our dataset and the large number of model–method combinations, we applied multiple comparison correction to control for false discovery rates (FDRs).^55^ We employed a one‐tailed significance test to determine whether for a higher‐performing model–method combination the larger F1 score was statistically significant compared to the score of a lower‐performing combination.

To assess the impact of RPE and FSPE, we utilized a mixed‐effects logistic regression model.^56^ This approach included a fixed‐effects term to evaluate the specific impact of RPE or FSPE, and a random‐effects term to account for variations across individual clinic notes. This analysis allowed us to determine whether the inclusion of RPE and FSPT led to statistically significant improvements in extraction performance.

Moreover, to draw a comparative analysis between the extraction capabilities of LLMs and current approaches in the field of medical information extraction, we incorporated the Medical Concept Annotation Tool (MedCAT),^57^ which has demonstrated superior performance to BioClinicalBERT^58^ in this domain.^57^ The version of MedCAT used in our study was fine‐tuned for epilepsy‐related information extraction. Subsequently, this fine‐tuned model was applied to the same set of 280 clinic letters used for the LLMs. The information extracted by MedCAT was then compared against the gold standard annotations to obtain metrics such as recall, precision, and F1 score.

In addition, to demonstrate the accuracy of human annotations, we treated one epileptologist as the gold standard while evaluating the performance of the other, ensuring that the evaluation avoids circular logic.

## 3 RESULTS

### 3.1 Dataset

Demographic and clinical characteristics of patients are summarized in Table 1. The dataset for our analysis comprised 280 annotated clinic letters of 280 individual patients.

The age distribution of the patients ranged from 18 to 80 years, with a notable concentration in the 21–30 and 31–40 age groups, accounting for 25.7% and 23.7% of the cohort, respectively. The median age of the patients was 36 years, with an average age of 38 years. Gender representation was nearly balanced, with females constituting 51%. The average number of current ASMs mentioned per clinic letter was 1.48, with a median of 1. We present relationships between current ASMs and associated symptoms in Appendix S2.

### 3.2 Extraction results

The Cohen's kappa score for the annotations provided by the two epileptologists is .84, which shows almost perfect agreement according to the Cohen's kappa score definition.^53^

The extraction results are shown in Table 2, where the entries are sorted by model and method, with the best‐performing results highlighted in bold for easier comparison. Given the finite size of our sample, we have also included the standard error for each metric (recall, precision, and F1) in Table 2. In addition, we have included another table in Appendix S3, which ranks the combinations by F1 scores in descending order. The extraction results allocated roles and provided few‐shot examples of the model and methods with optimal results from these four extraction tasks are shown in Table 3. We utilized a mixed‐effects logistic regression model to assess the impact of RPE and FSPE, and included the coefficients, which represent the magnitude of the impact that RPE and FSPE have on the model's performance, and *p*‐values in Table 3.

**TABLE 2:** Extraction performance and metric standard error (SE) for different LLMs and methods on all tasks.

Task	Model	Method	TP+FN	TP+FP	Recall	Recall SE	Precision	Precision SE	F1	F1 SE
Epilepsy types	Epileptologists	Manually	267	272	.**87**	.021	.**85**	.022	.**86**	.021
	Llama 2 7b	DE	258	277	.71	.028	.66	.028	.68	.028
	Llama 2 13b	DE	258	280	.**84**	.023	.**77**	.025	.**80**	.024
	Llama 3 8b	DE	258	285	.72	.028	.65	.028	.68	.028
	Mistral	DE	258	275	.45	.031	.42	.030	.43	.030
	Mixtral	DE	258	296	.53	.031	.47	.029	.50	.030
	Llama 2 7b	CDE	258	325	.75	.027	.60	.027	.67	.028
	Llama 2 13b	CDE	258	265	.54	.031	.53	.031	.54	.030
	Llama 3 8b	CDE	258	266	.57	.031	.55	.031	.56	.030
	Mistral	CDE	258	274	.67	.029	.64	.029	.65	.029
	Mixtral	CDE	258	303	.59	.031	.50	.029	.55	.030
	Llama 2 7b	SDE	258	274	.64	.030	.61	.029	.62	.029
	Llama 2 13b	SDE	258	262	.51	.031	.50	.031	.50	.030
	Llama 3 8b	SDE	258	240	.59	.031	.64	.031	.61	.029
	Mistral	SDE	258	281	.39	.030	.36	.029	.37	.029
	Mixtral	SDE	258	294	.60	.030	.52	.029	.56	.030
	Llama 2 7b	CSDE	258	289	.65	.030	.58	.029	.61	.029
	Llama 2 13b	CSDE	258	281	.48	.031	.44	.030	.46	.030
	Llama 3 8b	CSDE	258	249	.50	.031	.52	.032	.51	.030
	Mistral	CSDE	258	280	.62	.030	.57	.030	.60	.029
	Mixtral	CSDE	258	297	.72	.028	.63	.028	.67	.028
	MedCAT	Fine‐tunig	258	126	.38	.030	.75	.039	.50	.030
Seizure types	Epileptologists	Manually	267	265	.**78**	.025	.**78**	.025	.**78**	.025
	Llama 2 7b	DE	274	336	.71	.027	.58	.027	.64	.029
	Llama 2 13b	DE	274	275	.**76**	.026	.**76**	.026	.**76**	.026
	Llama 3 8b	DE	274	200	.51	.030	.70	.032	.59	.029
	Mistral	DE	274	278	.64	.029	.63	.029	.64	.029
	Mixtral	DE	274	268	.57	.030	.58	.030	.58	.029
	Llama 2 7b	CDE	274	405	.72	.027	.49	.025	.58	.029
	Llama 2 13b	CDE	274	251	.64	.029	.70	.029	.67	.028
	Llama 3 8b	CDE	274	221	.46	.030	.57	.033	.51	.030
	Mistral	CDE	274	273	.62	.029	.62	.029	.62	.029
	Mixtral	CDE	274	234	.41	.030	.48	.033	.44	.030
	Llama 2 7b	SDE	274	287	.55	.030	.53	.029	.54	.030
	Llama 2 13b	SDE	274	252	.53	.030	.58	.031	.55	.030
	Llama 3 8b	SDE	274	236	.54	.030	.63	.031	.58	.029
	Mistral	SDE	274	280	.51	.030	.50	.030	.50	.030
	Mixtral	SDE	274	304	.75	.026	.68	.027	.71	.027
	Llama 2 7b	CSDE	274	330	.62	.029	.51	.028	.56	.030
	Llama 2 13b	CSDE	274	260	.53	.030	.56	.031	.55	.030
	Llama 3 8b	CSDE	274	259	.54	.030	.57	.031	.55	.030
	Mistral	CSDE	274	281	.67	.028	.65	.028	.66	.028
	Mixtral	CSDE	274	314	.76	.026	.67	.027	.71	.027
	MedCAT	Fine‐tunig	274	147	.39	.029	.72	.037	.50	.030
Current ASMs	Epileptologists	Manually	394	395	.**95**	.011	.**95**	.011	.**95**	.013
	Llama 2 7b	DE	400	600	.95	.011	.63	.020	.76	.026
	Llama 2 13b	DE	400	440	.95	.011	.86	.017	.**90**	.018
	Llama 3 8b	DE	400	365	.71	.023	.78	.022	.74	.026
	Mistral	DE	400	447	.84	.018	.75	.020	.80	.024
	Mixtral	DE	400	398	.77	.021	.77	.021	.77	.025
	Llama 2 7b	SDE	400	465	.91	.014	.78	.019	.84	.022
	Llama 2 13b	SDE	400	92	.21	.020	.**92**	.028	.35	.029
	Llama 3 8b	SDE	400	415	.87	.017	.84	.018	.86	.021
	Mistral	SDE	400	272	.58	.025	.86	.021	.69	.028
	Mixtral	SDE	400	434	.**96**	.010	.88	.016	.**92**	.016
	MedCAT	Fine‐tunig	400	601	.88	.016	.59	.020	.70	.027
Associated symptoms	Epileptologists	Manually	70	39	.50	.060	.**90**	.048	.**64**	.029
	Llama 2 7b	DE	82	82	.60	.054	.**60**	.054	.60	.029
	Llama 2 13b	DE	82	555	.93	.028	.14	.015	.24	.026
	Llama 3 8b	DE	82	178	.70	.051	.32	.035	.44	.030
	Mistral	DE	82	146	.88	.036	.49	.041	.**63**	.029
	Mixtral	DE	82	241	.**95**	.024	.32	.030	.48	.030
	Llama 2 7b	SDE	82	51	.26	.048	.41	.069	.32	.028
	Llama 2 13b	SDE	82	459	.54	.055	.10	.014	.16	.022
	Llama 3 8b	SDE	82	136	.59	.054	.35	.041	.44	.030
	Mistral	SDE	82	72	.48	.055	.54	.059	.51	.030
	Mixtral	SDE	82	136	.79	.045	.48	.043	.60	.029
	MedCAT	Fine‐tunig	82	70	.45	.055	.53	.060	.49	.030

*Note*: “Professional Medical Information Extractor” (role1 in the table) and “Expert Epilepsy Neurologist” (role2 in the table). Bold content indicates the highest evaluation results. TP + FN = number of “positive” findings. TP + FP = number of findings predicted “positive”.

Abbreviations: CDE, Contextualized Direct Extraction; CSDE, Contextualized–Summarized Direct Extraction; DE, Direct Extraction; RPE, Role Prompting Extraction; SDE, Summarized Direct Extraction.

**TABLE 3:** Performance comparison of RPE and FSPE.

Model	Method	F1	Coefficient		*p*‐Value	Model	Method	F1	Coefficient	*p*‐Value
	Epilepsy types									
Llama 2 13b	DE	.80								
Llama 2 13b	DE RPE role1	.73	.012		.008	Llama 2 13b	DE FSPE 1	.15	.125	2.56 × 10^−12^
Llama 2 13b	DE RPE role2	.81	.012		.401	Llama 2 13b	DE FSPE 4	.18	.125	2.56 × 10^−12^
	Seizure types									
Llama 2 13b	DE	.76								
Llama 2 13b	DE RPE role1	.68	.02		.202	Llama 2 13b	DE FSPE 1	.17	.15	3.97 × 10^−10^
Llama 2 13b	DE RPE role2	.70	.018		.001	Llama 2 13b	DE FSPE 4	.18	.157	2.24 × 10^−11^
	Current ASMs									
Mixtral	SDE	.92								
Mixtral	SDE RPE role1	.89	.001		.671	Mixtral	SDE FSPE 1	.81	.003	8.85 × 10^−5^
Mixtral	SDE RPE role2	.87	.002		.004	Mixtral	SDE FSPE 4	.90	.002	.226
	Associated symptoms									
Llama 2 7b	DE	.60								
Llama 2 7b	DE RPE role1	.53	.001		.889	Llama 2 7b	DE FSPE 1	.31	.01	4.10 × 10^−9^
Llama 2 7b	DE RPE role2	.54	.007		.051	Llama 2 7b	DE FSPE 4	.27	.011	4.04 × 10^−14^

*Note*: Professional Medical Information Extractor (role1), Expert Epilepsy Neurologist (role 2), 1‐shot prompting (1), few‐shot prompting with four eaxmples (4).

Abbreviations: CDE, Contextualized Direct Extraction; CSDE, Contextualized–Summarized Direct Extraction; DE, Direct Extraction; FSPE, Few‐Shot Prompting Extraction; RPE, Role Prompting Extraction; SDE, Summarized Direct Extraction.

We performed pairwise comparisons of model–method combinations across four categories of extraction tasks, with all reported results adjusted for multiple comparisons to ensure statistical robustness. Specifically, we verified the robustness of our findings using the FDR correction via the Benjamini‐Hochberg procedure, which controls the false positive rate, ensuring statistical power while minimizing the likelihood of erroneous discoveries in multiple comparisons.^55^ A total of 594 comparison pairs were conducted, and all *p*‐value results after correction are provided in Appendix S4.

### 3.3 Extraction time

We also computed the average duration required for each model and method to extract individual pieces of information from a single clinic letter (Table 4).

**TABLE 4:** Average run time (seconds/clinic letter) for different LLMs and methods.

Model	Method	Epilepsy types	Seizure types	Current ASMs	Associated symptoms
Llama 2 7b	DE	7.7	9.7	6.2	7.8
Llama 2 7b	SDE	19.3	25.7	20.2	29.0
Llama 2 7b	CDE	9.3	10.4	–	–
Llama 2 7b	CSDE	19.4	26.8	–	–
Llama 2 13b	DE	7.6	8.0	10.7	6.4
Llama 2 13b	SDE	19.3	16.1	16.8	16.7
Llama 2 13b	CDE	9.0	9.0	–	–
Llama 2 13b	CSDE	18.8	15.7	–	–
Llama 3 8b	DE	20.2	21.6	23.0	26.7
Llama 3 8b	SDE	37.5	38.9	31.2	39.3
Llama 3 8b	CDE	19.7	20.0	–	–
Llama 3 8b	CSDE	37.6	38.8	–	–
Mistral	DE	3.5	3.1	10.0	4.4
Mistral	SDE	29.2	24.6	22.1	26.6
Mistral	CDE	3.3	3.0	–	–
Mistral	CSDE	30.0	24.9	–	–
Mixtral	DE	8.8	7.7	16.3	13.6
Mixtral	SDE	38.0	33.4	22.5	50.1
Mixtral	CDE	9.0	8.0	–	–
Mixtral	CSDE	37.9	33.4	–	–
Llama 2 13b	DE RPE role1	12.6	13.3	–	–
Llama 2 13b	DE RPE role2	14.2	13.5	–	–
Mixtral	SDE RPE role1	–	–	29.2	–
Mixtral	SDE RPE role2	–	–	29.7	–
Llama 2 7b	DE RPE role1	–	–	–	6.5
Llama 2 7b	DE RPE role2	–	–	–	6.1
Llama 2 13b	DE FSPE 1	9.8	10.7	–	–
Llama 2 13b	DE FSPE 4	9.6	11.3	–	–
Mixtral	SDE FSPE 1	–	–	19.4	–
Mixtral	SDE FSPE 4	–	–	20.8	–
Llama 2 7b	DE FSPE 1	–	–	–	4.4
Llama 2 7b	DE FSPE 4	–	–	–	4.2
Epileptologists	Manually	110.0			

*Note*: Professional Medical Information Extractor(role1), Expert Epilepsy Neurologist(role 2), 1‐shot prompting (1), few‐shot prompting with 4 examples (4).

Abbreviations: DE, Direct Extraction; CDE, Contextualized‐Direct Extraction; CSDE, Contextualized‐Summarized‐Direct Extraction; FSPE, Few‐Shot Prompting Extraction; RPE, Role Prompting Extraction; SDE, Summarized‐Direct Extraction.

## 4 DISCUSSION

The analysis of various models highlights that Llama 2 13b consistently outperforms other LLMs based on the F1 score, demonstrating high performance across most tasks, particularly in epilepsy type, seizure type, and current ASM extraction. Similarly, Mixtral exhibits commendable performance with its F1 scores typically ranking second highest among those of other LLMs. Llama 2 7b and Mistral show uneven performance. They exhibit reliability in specific contexts such as associated symptom extraction; however, they do not uniformly achieve the peak performance of their counterparts, suggesting potential constraints in accommodating the nuances of diverse tasks. Although Llama 3 8b is the latest Llama‐series model, its performance consistently falls short of surpassing its predecessors in the Llama 2 series. Despite fine‐tuning on epilepsy‐related text, MedCAT underperforms compared to LLMs, highlighting the advantages of LLMs over current approaches.

Comparatively, in extraction method assessments, DE consistently maintains a high standard, particularly in tasks such as current ASMs, where the information extraction is relatively straightforward with minimal ambiguity and variations in terminology. SDE shows variable performance, with the text summarization ability of the LLM directly influencing its effectiveness. It is obvious that imprecise or incomplete summaries can adversely affect the outcome, regardless of the accuracy of subsequent extraction chains. The introduction of CDE and CSDE in the epilepsy type and seizure type extraction tasks did not result in a significant impact on the outcomes.

In the RPE method, we assigned two distinct roles to LLMs: “Professional Medical Information Extractor” and “Expert Epilepsy Neurologist,” with the former being a machine and the latter a human. The findings indicate that the RPE method impacts extraction outcomes, yet in our task, such impacts are typically not positive, although the performance reduction is marginal. In comparisons between the two roles, the “Expert Epilepsy Neurologist” role slightly outperforms the “Professional Medical Information Extractor” in extracting epilepsy type, seizure type, and associated symptoms. The mixed‐effects logistic regression model indicated that the performance of RPE for improving F1 scores were minimal, and the statistical significance was inconsistent, with only some cases showing significant results. In the FSPE method, the four‐example (few‐shot) prompting does not perform better than the one‐example (1‐shot) prompting. We compared the outcomes of providing LLMs with either a single set or four sets of “input–output” examples. The results demonstrate that for complex tasks such as medical information extraction, which involves extracting multiple categories, FSPE does not effectively enhance performance. In fact, for certain tasks, such as epilepsy or seizure type extraction, it performs poorly. This inadequacy could be attributed to the complexity of the extraction, which requires a significant number of examples for effective performance. However, a large volume of examples can lead to difficulties in LLM processing and even result in memory overflow. Moreover, we observed that the difference in effectiveness between using one‐shot and four‐shot examples is minimal. The mixed‐effects logistic regression model incorporating FSPE demonstrated that the inclusion of FSPE consistently led to a decrease in F1 scores, and these differences were statistically significant across all cases. This contrasts significantly with the findings of a previous study,^41^ which observed marked differences between zero‐shot, one‐shot, and few‐shot prompting in both free‐form generation tasks and multiple choice tasks, indicating that the approach is ineffective for the medical information extraction task in this study.

Across various extraction tasks, Llama 2 13b DE excels in recall, precision, and F1 scores for epilepsy type and seizure type extractions. Mixtral SDE attains the highest F1 score in current ASMs extraction, but is only .02 points higher than Llama 2 13b DE. In the extraction of associated symptoms, Llama 2 7b DE achieves the highest precision in all LLMs, with higher recall than epileptologist annotations. In addition, we specifically examined these best‐performing LLM–method combination for each extraction task, and each F1 score was also significantly higher than those of other LLM–method combinations.

The findings suggest that LLMs, particularly Llama b2 13b, exhibit superior extraction capabilities in tasks such as epilepsy type, current ASMs, and seizure type. Although the results for associated symptom extraction are not as high, this may be attributed to the infrequent mention of associated symptoms in clinic letters and sometimes indirect references. Overall, LLMs outperform MedCAT in epilepsy‐related information extraction from unstructured clinic letters across various tasks. Notably, despite extensive training and fine‐tuning on epilepsy‐related information extraction, MedCAT is surpassed by LLMs, which have not undergone any fine‐tuning.

By comparing discrepancies between LLMs and human annotations, we have identified some limitations of LLMs in extracting epilepsy‐related information. Epileptologists consistently achieved the highest F1 scores, and their F1 scores were significantly higher than those of all model‐based extraction methods. Epileptologist annotators achieved a very high F1 score of .95 for ASMs, and the best F1 score among the LLMs tested was .92 for Mixtral, which is also high. Nevertheless, this comparison still indicates a difference in performance of 3 percentage points, which highlights the current limitations of LLMs. We believe that in this case the lower performance of the LLMs is due to two key factors. First, LLMs are trained on large general corpora, whereas our two epileptologist annotators are highly trained in the specific domain of epilepsy. Second, although extraction tasks such as ASMs involve standardized terminology, there are occasional instances of variations in documentation style, abbreviations, and implicit references—where ASMs are inferred rather than explicitly stated. Although these cases are easily interpreted by human experts through clinical reasoning, they remain challenging for LLMs. This explains why ASM extraction generally performs well but still falls short compared to epileptologists. By contrast, the epileptologist annotators can analyze vague or imprecise language and make a judgment on how it could be interpreted in this context. The limitations of LLMs are even more obvious for epilepsy type and seizure type extractions. Whereas epileptologist annotators achieved an F1 score of .86 and .78, respectively, the best performing LLM for these two categories, Llama 2 13b, recorded F1 scores of a much lower .80 and .76, respectively. This difference in performance of 3–6 percentage points highlights even more the above two factors. The language used in clinic letters for epilepsy type and seizure type is typically even more specialized than that for ASMs. However, the situation is more complex when it comes to identifying associated symptoms. Epileptologist annotators posted a poor performance of .64 for F1, revealing how difficult it is for epileptologists to extract this information from clinic letters. Low recall (.50) confirmed that manual extraction often overlooks some associated symptoms that need to be identified. Nevertheless, the best performing LLM for this category, Mistral, recorded an F1 score of .63, 1 percentage point behind. Our conclusion, therefore, is that even though LLMs are not great for extracting associated symptoms, their performance is broadly in line with epileptologists, who also struggled with this category.

Regarding run time, it is apparent that the more chains involved in LangChain, the longer the processing time. Extractions involving summarization are the most time‐consuming. The LLM parameter size directly correlates with longer run time for the same extraction task and extraction method. The times are considered acceptable, particularly when processing large volumes of text in parallel, offering a more efficient alternative to manual extraction. The introduction of RPE and FSPE methods exhibits minimal impact on the execution time. Similarly, the different roles do not significantly affect the duration of the process. Furthermore, there is no notable difference in the execution time between using one example and four examples.

Based on our findings, although the performance of LLMs has not yet reached the requirement for fully autonomous clinical use, their accuracy could potentially support certain downstream research in the future. In large‐scale ML applications, a certain level of inaccuracy is often tolerable, and minor extraction errors are unlikely to significantly impact overall model performance.^59^ In this context, LLMs can facilitate the analysis of large clinical datasets, thereby advancing broader research applications. However, achieving unsupervised clinical use with direct impact on patient outcomes would require more rigorous validation and a higher degree of accuracy. Given that clinical decisions have direct implications for patient health and treatment efficacy, any extraction errors could pose potential risks. Therefore, we recommend that, in clinical practice, LLM‐derived information should be used as supplementary data for clinicians rather than as a sole basis for decision‐making. This approach leverages the processing efficiency of LLMs while ensuring the rigor and safety required in clinical decision‐making.

This study explores and compares the potential of different LLMs and methods in extracting epilepsy‐related information from unstructured clinic letters. Our findings not only demonstrate the capabilities of LLMs in handling complex medical data but also offer new perspectives and technical approaches to address the broader challenge of extracting information from unstructured data.^60^ Although LLMs have not yet reached the level required for fully autonomous clinical use, their accuracy and efficiency suggest significant potential for supporting clinicians in identifying key medical information, thereby reducing the time burden of manual record review and improving clinical workflow. In large‐scale research applications, LLMs can facilitate the extraction and standardization of medical information from vast amounts of unstructured data, enabling more efficient data‐driven research and epidemiologic analysis.^61^

Despite these advantages, this study finds that LLMs still exhibit limitations, particularly in handling implicit references and nuanced contextual understanding, which currently restrict their unsupervised use in clinical decision‐making. These challenges highlight the importance of human oversight when integrating LLMs into clinical workflows.^62^ Nevertheless, rapid advancements in LLM development suggest that these limitations may be mitigated in future iterations of models or techniques.^63^ Fine‐tuning LLMs on domain‐specific datasets could further enhance their reliability and transparency, making them more viable for clinical applications.^64^ The introduction of these new methods and technologies not only provides robust data for research on complex diseases like epilepsy but also paves the way for advancing personalized treatment and artificial intelligence–assisted clinical decision‐making.^65^, ^66^

## AUTHOR CONTRIBUTIONS

**Shichao Fang:** contributed to the conceptualization, methodology, software, validation, formal analysis, investigation, data curation, writing – original draft, writing review and editing, and visualization. **Ben Holgate:** contributed to the methodology, formal analysis, validation, and writing – review and editing. **Anthony Shek:** contributed to the methodology, investigation, software, data curation, and writing – review and editing. **Joel S. Winston:** contributed to the validation and writing – review and editing. **Matthew McWilliam:** contributed to the validation and writing – review and editing. **Pedro F. Viana:** contributed to the validation and writing – review and editing. **James T. Teo:** contributed to the methodology, software, resources, data curation, and writing – review and editing. **Mark P. Richardson:** contributed to the conceptualization, validation, resources, writing – original draft, writing review and editing, supervision, project administration, and funding acquisition.

## CONFLICT OF INTEREST STATEMENT

M.P.R. has received research funding from Epilepsy Research Institute UK. J.T.T. has received research funding from Innovate UK, Office of Life Sciences, Health Data Research UK, and National Institutes of Health Research UK. J.T.T. is a director of CogStack Ltd. We confirm that we have read the Journal's position on issues involved in ethical publication and affirm that this report is consistent with those guidelines.

## Supporting information


Appendix S1.

## Data Availability

The data used in this study are from King's College Hospital NHS Foundation Trust. The original code used in this work can be found in https://github.com/scfang6/extracting_information_using_LLMs.
