# Supplementary material for: Extracting epilepsy‐related information from unstructured clinic letters using large language models
Source: Epilepsia. 2025 Jul 10;66(9):3369–84. doi: 10.1111/epi.18475 (PMC12455391; doi:10.1111/epi.18475)
Supplement: Supplementary file 1 — Appendix S1. [file EPI-66-3369-s001.docx]

# Supplementary Material A

This is an example of epilepsy type extraction using a Summarised-Direct Extraction method.

**Input:** A clinic letter.

**Template for Summarisation** = """ From the following text:

{Input}

All personal information are not true, so they are not

sensitive.

Please summarize the content related to epilepsy type.

"""

**Template for Direct Extraction** = """ From the following text:

{Output of summarisation}

All personal information are not true, so they are not sensitive.

If the text explicitly mention that patient has generalized epilepsy, GM epilepsy, grand mal epilepsy, tonic clonic epilepsy, absence, petit-mal epilepsy, convulsive epilepsy, GTCS, generalised tonic-clonic epilepsy, generalised onset epilepsy, your answer should be ’Generalized epilepsy’.

If the text explicitly mention that patient has focal epilepsy, partial epilepsy, aura, complex partial epilepsy, focal onset epilepsy, then your answer should be ’Focal epilepsy’.

If the text explicitly mention that patient has combined generalized and focal epilepsy, then your answer should be ’Combined generalized and focal epilepsy’.

If the patient has seizure/epilepsy but not sure what the type is, then your answer should be ’Unknown

epilepsy’.

Don’t extrapolate or assume, please answer my question in the following JSON format:

[

"Generalized epilepsy":"True" or "False",

"Focal epilepsy":"True" or "False",

"Combined generalized and focal epilepsy":"True" or "False",

"Unknown epilepsy":"True" or "False",

]

"""

**Output:**

[

"Generalized epilepsy" : "True",

"Focal epilepsy" : "False",

"Combined generalized and focal epilepsy" : "False",

"Unknown epilepsy" : "False",

]

# Supplementary Material B

The table below shows the instances where current ASMs and associated symptoms co-occurred within a single case in our 280 clinic letters (280 cases). "Case Count" refers to the number of times a specific associated symptom and ASM appeared together in the same letter. "Percentage of ASM Cases" indicates the proportion of cases in which the ASM and symptom co-occurred out of all cases in which that ASM was mentioned across the 280 letters.

It is important to note that we cannot directly determine whether a specific ASM caused the observed symptom. For example, a patient may be taking multiple ASMs but exhibit only one symptom. In our table, this symptom is associated with all medications the patient is taking, though some of these ASMs may not actually contribute to the symptom. However, a higher co-occurrence percentage may suggest a stronger association between a specific medication and the symptom.

| **Symptoms** | **ASMs** | **Case Count** | **Percentage of ASM Cases** |
| --- | --- | --- | --- |
| anxiety | Carbamazepine | 3 | 5% |
|  | Clobazam | 2 | 5% |
|  | Lacosamide | 2 | 9% |
|  | Lamotrigine | 4 | 7% |
|  | Levetiracetam | 4 | 6% |
|  | Oxcarbazepine | 1 | 14% |
|  | Perampanel | 1 | 17% |
|  | Sodium Valproate | 4 | 8% |
|  | Zonisamide | 1 | 6% |
| depression | Carbamazepine | 6 | 10% |
|  | Clobazam | 4 | 11% |
|  | Lacosamide | 2 | 9% |
|  | Lamotrigine | 3 | 5% |
|  | Levetiracetam | 5 | 8% |
|  | Oxcarbazepine | 1 | 14% |
|  | Perampanel | 2 | 33% |
|  | Sodium Valproate | 5 | 9% |
|  | Topiramate | 1 | 5% |
|  | Zonisamide | 2 | 12% |
| dizziness | Clobazam | 1 | 3% |
|  | Lacosamide | 1 | 5% |
|  | Lamotrigine | 2 | 3% |
| headache | Carbamazepine | 6 | 10% |
|  | Clobazam | 4 | 11% |
|  | Lamotrigine | 3 | 5% |
|  | Levetiracetam | 3 | 5% |
|  | Phenytoin | 1 | 7% |
|  | Sodium Valproate | 3 | 6% |
|  | Zonisamide | 2 | 12% |
| lethargy | Acetazolamide | 1 | 50% |
|  | Carbamazepine | 4 | 7% |
|  | Clobazam | 5 | 13% |
|  | Clonazepam | 1 | 20% |
|  | Lacosamide | 1 | 5% |
|  | Lamotrigine | 7 | 12% |
|  | Levetiracetam | 8 | 13% |
|  | Oxcarbazepine | 1 | 14% |
|  | Perampanel | 1 | 17% |
|  | Phenytoin | 1 | 7% |
|  | Pregabalin | 1 | 17% |
|  | Retigabine | 1 | 100% |
|  | Rufinamide | 1 | 33% |
|  | Sodium Valproate | 3 | 6% |
|  | Topiramate | 1 | 5% |
|  | Zonisamide | 4 | 24% |
| nausea | Carbamazepine | 1 | 2% |
|  | Clobazam | 1 | 3% |
|  | Lamotrigine | 1 | 2% |
| rash | Lacosamide | 1 | 5% |

# Supplementary Material C

We have sorted the Table 2 from the highest F1 to the lowest in the following table.

| Model | Method | TP+FN | TP+FP | Recall | Recall SE | Precision | Precision SE | F1 | F1 SE |
| --- | --- | --- | --- | --- | --- | --- | --- | --- | --- |
| Epilepsy types | | | | | | | | | |
| Epileptologists | Manually | 267 | 272 | **0.87** | 0.021 | **0.85** | 0.022 | **0.86** | 0.021 |
| Llama2 13b | DE | 258 | 280 | **0.84** | 0.023 | **0.77** | 0.025 | **0.8** | 0.024 |
| Llama2 7b | DE | 258 | 277 | 0.71 | 0.028 | 0.66 | 0.028 | 0.68 | 0.028 |
| Llama3 8b | DE | 258 | 285 | 0.72 | 0.028 | 0.65 | 0.028 | 0.68 | 0.028 |
| Llama2 7b | CDE | 258 | 325 | 0.75 | 0.027 | 0.6 | 0.027 | 0.67 | 0.028 |
| Mixtral | CSDE | 258 | 297 | 0.72 | 0.028 | 0.63 | 0.028 | 0.67 | 0.028 |
| Mistral | CDE | 258 | 274 | 0.67 | 0.029 | 0.64 | 0.029 | 0.65 | 0.029 |
| Llama2 7b | SDE | 258 | 274 | 0.64 | 0.03 | 0.61 | 0.029 | 0.62 | 0.029 |
| Llama3 8b | SDE | 258 | 240 | 0.59 | 0.031 | 0.64 | 0.031 | 0.61 | 0.029 |
| Llama2 7b | CSDE | 258 | 289 | 0.65 | 0.03 | 0.58 | 0.029 | 0.61 | 0.029 |
| Mistral | CSDE | 258 | 280 | 0.62 | 0.03 | 0.57 | 0.03 | 0.6 | 0.029 |
| Llama3 8b | CDE | 258 | 266 | 0.57 | 0.031 | 0.55 | 0.031 | 0.56 | 0.03 |
| Mixtral | SDE | 258 | 294 | 0.6 | 0.03 | 0.52 | 0.029 | 0.56 | 0.03 |
| Mixtral | CDE | 258 | 303 | 0.59 | 0.031 | 0.5 | 0.029 | 0.55 | 0.03 |
| Llama2 13b | CDE | 258 | 265 | 0.54 | 0.031 | 0.53 | 0.031 | 0.54 | 0.03 |
| Llama3 8b | CSDE | 258 | 249 | 0.5 | 0.031 | 0.52 | 0.032 | 0.51 | 0.03 |
| Mixtral | DE | 258 | 296 | 0.53 | 0.031 | 0.47 | 0.029 | 0.5 | 0.03 |
| Llama2 13b | SDE | 258 | 262 | 0.51 | 0.031 | 0.5 | 0.031 | 0.5 | 0.03 |
| MedCAT | Fine-tuning | 258 | 126 | 0.38 | 0.03 | 0.75 | 0.039 | 0.5 | 0.03 |
| Llama2 13b | CSDE | 258 | 281 | 0.48 | 0.031 | 0.44 | 0.03 | 0.46 | 0.03 |
| Mistral | DE | 258 | 275 | 0.45 | 0.031 | 0.42 | 0.03 | 0.43 | 0.03 |
| Mistral | SDE | 258 | 281 | 0.39 | 0.03 | 0.36 | 0.029 | 0.37 | 0.029 |
| Seizure types | | | | | | | | | |
| Epileptologists | Manually | 267 | 265 | **0.78** | 0.025 | **0.78** | 0.025 | **0.78** | 0.025 |
| Llama2 13b | DE | 274 | 275 | **0.76** | 0.026 | **0.76** | 0.026 | **0.76** | 0.026 |
| Mixtral | SDE | 274 | 304 | 0.75 | 0.026 | 0.68 | 0.027 | 0.71 | 0.027 |
| Mixtral | CSDE | 274 | 314 | 0.76 | 0.026 | 0.67 | 0.027 | 0.71 | 0.027 |
| Llama2 13b | CDE | 274 | 251 | 0.64 | 0.029 | 0.7 | 0.029 | 0.67 | 0.028 |
| Mistral | CSDE | 274 | 281 | 0.67 | 0.028 | 0.65 | 0.028 | 0.66 | 0.028 |
| Llama2 7b | DE | 274 | 336 | 0.71 | 0.027 | 0.58 | 0.027 | 0.64 | 0.029 |
| Mistral | DE | 274 | 278 | 0.64 | 0.029 | 0.63 | 0.029 | 0.64 | 0.029 |
| Mistral | CDE | 274 | 273 | 0.62 | 0.029 | 0.62 | 0.029 | 0.62 | 0.029 |
| Llama3 8b | DE | 274 | 200 | 0.51 | 0.03 | 0.7 | 0.032 | 0.59 | 0.029 |
| Mixtral | DE | 274 | 268 | 0.57 | 0.03 | 0.58 | 0.03 | 0.58 | 0.029 |
| Llama2 7b | CDE | 274 | 405 | 0.72 | 0.027 | 0.49 | 0.025 | 0.58 | 0.029 |
| Llama3 8b | SDE | 274 | 236 | 0.54 | 0.03 | 0.63 | 0.031 | 0.58 | 0.029 |
| Llama2 7b | CSDE | 274 | 330 | 0.62 | 0.029 | 0.51 | 0.028 | 0.56 | 0.03 |
| Llama2 13b | SDE | 274 | 252 | 0.53 | 0.03 | 0.58 | 0.031 | 0.55 | 0.03 |
| Llama2 13b | CSDE | 274 | 260 | 0.53 | 0.03 | 0.56 | 0.031 | 0.55 | 0.03 |
| Llama3 8b | CSDE | 274 | 259 | 0.54 | 0.03 | 0.57 | 0.031 | 0.55 | 0.03 |
| Llama2 7b | SDE | 274 | 287 | 0.55 | 0.03 | 0.53 | 0.029 | 0.54 | 0.03 |
| Llama3 8b | CDE | 274 | 221 | 0.46 | 0.03 | 0.57 | 0.033 | 0.51 | 0.03 |
| Mistral | SDE | 274 | 280 | 0.51 | 0.03 | 0.5 | 0.03 | 0.5 | 0.03 |
| MedCAT | Fine-tuning | 274 | 147 | 0.39 | 0.029 | 0.72 | 0.037 | 0.5 | 0.03 |
| Mixtral | CDE | 274 | 234 | 0.41 | 0.03 | 0.48 | 0.033 | 0.44 | 0.03 |
| Current ASMs | | | | | | | | | |
| Epileptologists | Manually | 394 | 395 | **0.95** | 0.011 | **0.95** | 0.011 | **0.95** | 0.013 |
| Mixtral | SDE | 400 | 434 | **0.96** | 0.01 | 0.88 | 0.016 | **0.92** | 0.016 |
| Llama2 13b | DE | 400 | 440 | 0.95 | 0.011 | 0.86 | 0.017 | **0.9** | 0.018 |
| Llama3 8b | SDE | 400 | 415 | 0.87 | 0.017 | 0.84 | 0.018 | 0.86 | 0.021 |
| Llama2 7b | SDE | 400 | 465 | 0.91 | 0.014 | 0.78 | 0.019 | 0.84 | 0.022 |
| Mistral | DE | 400 | 447 | 0.84 | 0.018 | 0.75 | 0.02 | 0.8 | 0.024 |
| Mixtral | DE | 400 | 398 | 0.77 | 0.021 | 0.77 | 0.021 | 0.77 | 0.025 |
| Llama2 7b | DE | 400 | 600 | 0.95 | 0.011 | 0.63 | 0.02 | 0.76 | 0.026 |
| Llama3 8b | DE | 400 | 365 | 0.71 | 0.023 | 0.78 | 0.022 | 0.74 | 0.026 |
| MedCAT | Fine-tuning | 400 | 601 | 0.88 | 0.016 | 0.59 | 0.02 | 0.7 | 0.027 |
| Mistral | SDE | 400 | 272 | 0.58 | 0.025 | 0.86 | 0.021 | 0.69 | 0.028 |
| Llama2 13b | SDE | 400 | 92 | 0.21 | 0.02 | **0.92** | 0.028 | 0.35 | 0.029 |
| Associated symptoms | | | | | | | | | |
| Epileptologists | Manually | 70 | 39 | 0.5 | 0.06 | **0.9** | 0.048 | **0.64** | 0.029 |
| Mistral | DE | 82 | 146 | 0.88 | 0.036 | 0.49 | 0.041 | **0.63** | 0.029 |
| Llama2 7b | DE | 82 | 82 | 0.6 | 0.054 | **0.6** | 0.054 | 0.6 | 0.029 |
| Mixtral | SDE | 82 | 136 | 0.79 | 0.045 | 0.48 | 0.043 | 0.6 | 0.029 |
| Mistral | SDE | 82 | 72 | 0.48 | 0.055 | 0.54 | 0.059 | 0.51 | 0.03 |
| MedCAT | Fine-tuning | 82 | 70 | 0.45 | 0.055 | 0.53 | 0.06 | 0.49 | 0.03 |
| Mixtral | DE | 82 | 241 | **0.95** | 0.024 | 0.32 | 0.03 | 0.48 | 0.03 |
| Llama3 8b | DE | 82 | 178 | 0.7 | 0.051 | 0.32 | 0.035 | 0.44 | 0.03 |
| Llama3 8b | SDE | 82 | 136 | 0.59 | 0.054 | 0.35 | 0.041 | 0.44 | 0.03 |
| Llama2 7b | SDE | 82 | 51 | 0.26 | 0.048 | 0.41 | 0.069 | 0.32 | 0.028 |
| Llama2 13b | DE | 82 | 555 | 0.93 | 0.028 | 0.14 | 0.015 | 0.24 | 0.026 |
| Llama2 13b | SDE | 82 | 459 | 0.54 | 0.055 | 0.1 | 0.014 | 0.16 | 0.022 |

*Note:* Direct Extraction (DE), Summarised-Direct Extraction (SDE), Contextualised-Direct Extraction (CDE), Contextualised-Summarised-Direct Extraction (CSDE). ‘Professional Medical Information Extractor’ (role1 in the table) and ‘Expert Epilepsy Neurologist’ (role2 in thet table). Bold content indicates the highest evaluation results. TP+FN= number of “positive” findings. TP+FP= number of findings predicted “positive”.

# Supplementary Material D

The four tables provided present the statistical comparison results for the four extraction tasks. In each pairwise comparison, “Model & Method 1” represents the model-method combination with a higher F1 score in the pair, while “Model & Method 2” corresponds to the combination with a lower F1 score. The reported p-values have been adjusted for multiple comparisons to ensure.

Corrected statistical comparison results of epilepsy type extraction

| Model&Method 1 | Model&Method 2 | Corrected p-value | Significant(p<0.05) |
| --- | --- | --- | --- |
| Epileptologists_Manually | Llama2_7b_DE | 7.44X10-24 | TRUE |
| Epileptologists_Manually | Llama2_13b_DE | 6.01X10-26 | TRUE |
| Epileptologists_Manually | Llama3_8b_DE | 5.50X10-38 | TRUE |
| Epileptologists_Manually | Mistral_DE | 7.10X10-147 | TRUE |
| Epileptologists_Manually | Mixtral_DE | 7.37X10-117 | TRUE |
| Epileptologists_Manually | Llama2_7b_CDE | 7.47X10-22 | TRUE |
| Epileptologists_Manually | Llama2_13b_CDE | 4.17X10-82 | TRUE |
| Epileptologists_Manually | Llama3_8b_CDE | 1.56X10-53 | TRUE |
| Epileptologists_Manually | Mistral_CDE | 4.78X10-61 | TRUE |
| Epileptologists_Manually | Mixtral_CDE | 3.00X10-80 | TRUE |
| Epileptologists_Manually | Llama2_7b_SDE | 9.46X10-20 | TRUE |
| Epileptologists_Manually | Llama2_13b_SDE | 2.74X10-31 | TRUE |
| Epileptologists_Manually | Llama3_8b_SDE | 7.98X10-41 | TRUE |
| Epileptologists_Manually | Mistral_SDE | 8.67X10-111 | TRUE |
| Epileptologists_Manually | Mixtral_SDE | 6.06X10-89 | TRUE |
| Epileptologists_Manually | Llama2_7b_CSDE | 3.12X10-27 | TRUE |
| Epileptologists_Manually | Llama2_13b_CSDE | 6.80X10-36 | TRUE |
| Epileptologists_Manually | Llama3_8b_CSDE | 3.15X10-52 | TRUE |
| Epileptologists_Manually | Mistral_CSDE | 3.13X10-21 | TRUE |
| Epileptologists_Manually | Mixtral_CSDE | 6.66X10-42 | TRUE |
| Epileptologists_Manually | MedCAT_Fine-tuning | 7.92X10-83 | TRUE |
| Llama2_13b_DE | Llama2_7b_DE | 1.20X10-03 | TRUE |
| Llama3_8b_DE | Llama2_7b_DE | 9.61X10-08 | TRUE |
| Llama2_7b_DE | Mistral_DE | 3.00X10-40 | TRUE |
| Llama2_7b_DE | Mixtral_DE | 4.09X10-34 | TRUE |
| Llama2_7b_DE | Llama2_7b_CDE | 2.31X10-01 | FALSE |
| Llama2_7b_DE | Llama2_13b_CDE | 9.63X10-18 | TRUE |
| Llama2_7b_DE | Llama3_8b_CDE | 2.69X10-05 | TRUE |
| Llama2_7b_DE | Mistral_CDE | 5.18X10-15 | TRUE |
| Llama2_7b_DE | Mixtral_CDE | 2.46X10-14 | TRUE |
| Llama2_7b_DE | Llama2_7b_SDE | 4.22X10-01 | FALSE |
| Llama2_7b_DE | Llama2_13b_SDE | 8.51X10-02 | FALSE |
| Llama2_7b_DE | Llama3_8b_SDE | 7.09X10-09 | TRUE |
| Llama2_7b_DE | Mistral_SDE | 4.94X10-27 | TRUE |
| Llama2_7b_DE | Mixtral_SDE | 7.36X10-22 | TRUE |
| Llama2_7b_DE | Llama2_7b_CSDE | 2.79X10-01 | FALSE |
| Llama2_7b_DE | Llama2_13b_CSDE | 5.79X10-02 | FALSE |
| Llama2_7b_DE | Llama3_8b_CSDE | 4.21X10-09 | TRUE |
| Llama2_7b_DE | Mistral_CSDE | 3.29X10-01 | FALSE |
| Llama2_7b_DE | Mixtral_CSDE | 3.35X10-03 | TRUE |
| Llama2_7b_DE | MedCAT_Fine-tuning | 2.23X10-11 | TRUE |
| Llama2_13b_DE | Llama3_8b_DE | 1.50X10-06 | TRUE |
| Llama2_13b_DE | Mistral_DE | 1.04X10-36 | TRUE |
| Llama2_13b_DE | Mixtral_DE | 3.37X10-25 | TRUE |
| Llama2_13b_DE | Llama2_7b_CDE | 1.86X10-05 | TRUE |
| Llama2_13b_DE | Llama2_13b_CDE | 3.44X10-08 | TRUE |
| Llama2_13b_DE | Llama3_8b_CDE | 6.85X10-03 | TRUE |
| Llama2_13b_DE | Mistral_CDE | 7.03X10-04 | TRUE |
| Llama2_13b_DE | Mixtral_CDE | 5.65X10-15 | TRUE |
| Llama2_13b_DE | Llama2_7b_SDE | 1.90X10-06 | TRUE |
| Llama2_13b_DE | Llama2_13b_SDE | 7.68X10-04 | TRUE |
| Llama2_13b_DE | Llama3_8b_SDE | 1.62X10-07 | TRUE |
| Llama2_13b_DE | Mistral_SDE | 3.25X10-17 | TRUE |
| Llama2_13b_DE | Mixtral_SDE | 7.00X10-15 | TRUE |
| Llama2_13b_DE | Llama2_7b_CSDE | 1.96X10-04 | TRUE |
| Llama2_13b_DE | Llama2_13b_CSDE | 1.97X10-02 | TRUE |
| Llama2_13b_DE | Llama3_8b_CSDE | 3.90X10-02 | TRUE |
| Llama2_13b_DE | Mistral_CSDE | 3.26X10-02 | TRUE |
| Llama2_13b_DE | Mixtral_CSDE | 2.17X10-03 | TRUE |
| Llama2_13b_DE | MedCAT_Fine-tuning | 8.80X10-12 | TRUE |
| Llama3_8b_DE | Mistral_DE | 7.52X10-16 | TRUE |
| Llama3_8b_DE | Mixtral_DE | 9.20X10-09 | TRUE |
| Llama3_8b_DE | Llama2_7b_CDE | 4.29X10-05 | TRUE |
| Llama3_8b_DE | Llama2_13b_CDE | 1.83X10-03 | TRUE |
| Llama3_8b_DE | Llama3_8b_CDE | 3.44X10-02 | TRUE |
| Llama3_8b_DE | Mistral_CDE | 4.20X10-05 | TRUE |
| Llama3_8b_DE | Mixtral_CDE | 5.46X10-09 | TRUE |
| Llama3_8b_DE | Llama2_7b_SDE | 1.19X10-02 | TRUE |
| Llama3_8b_DE | Llama2_13b_SDE | 1.80X10-02 | TRUE |
| Llama3_8b_DE | Llama3_8b_SDE | 3.32X10-01 | FALSE |
| Llama3_8b_DE | Mistral_SDE | 1.22X10-10 | TRUE |
| Llama3_8b_DE | Mixtral_SDE | 5.12X10-08 | TRUE |
| Llama3_8b_DE | Llama2_7b_CSDE | 9.53X10-08 | TRUE |
| Llama3_8b_DE | Llama2_13b_CSDE | 1.12X10-01 | FALSE |
| Llama3_8b_DE | Llama3_8b_CSDE | 1.56X10-01 | FALSE |
| Llama3_8b_DE | Mistral_CSDE | 1.57X10-08 | TRUE |
| Llama3_8b_DE | Mixtral_CSDE | 2.98X10-01 | FALSE |
| Llama3_8b_DE | MedCAT_Fine-tuning | 1.63X10-07 | TRUE |
| Mixtral_DE | Mistral_DE | 1.46X10-03 | TRUE |
| Llama2_7b_CDE | Mistral_DE | 5.80X10-34 | TRUE |
| Llama2_13b_CDE | Mistral_DE | 5.11X10-11 | TRUE |
| Llama3_8b_CDE | Mistral_DE | 7.33X10-12 | TRUE |
| Mistral_CDE | Mistral_DE | 3.62X10-10 | TRUE |
| Mixtral_CDE | Mistral_DE | 3.44X10-04 | TRUE |
| Llama2_7b_SDE | Mistral_DE | 4.52X10-46 | TRUE |
| Llama2_13b_SDE | Mistral_DE | 2.56X10-31 | TRUE |
| Llama3_8b_SDE | Mistral_DE | 4.22X10-15 | TRUE |
| Mistral_DE | Mistral_SDE | 4.92X10-02 | TRUE |
| Mixtral_SDE | Mistral_DE | 1.76X10-03 | TRUE |
| Llama2_7b_CSDE | Mistral_DE | 6.73X10-35 | TRUE |
| Llama2_13b_CSDE | Mistral_DE | 8.06X10-25 | TRUE |
| Llama3_8b_CSDE | Mistral_DE | 7.85X10-18 | TRUE |
| Mistral_CSDE | Mistral_DE | 4.87X10-42 | TRUE |
| Mixtral_CSDE | Mistral_DE | 2.32X10-16 | TRUE |
| MedCAT_Fine-tuning | Mistral_DE | 2.89X10-14 | TRUE |
| Llama2_7b_CDE | Mixtral_DE | 7.04X10-23 | TRUE |
| Llama2_13b_CDE | Mixtral_DE | 1.78X10-02 | TRUE |
| Llama3_8b_CDE | Mixtral_DE | 4.90X10-06 | TRUE |
| Mistral_CDE | Mixtral_DE | 8.36X10-10 | TRUE |
| Mixtral_CDE | Mixtral_DE | 9.57X10-07 | TRUE |
| Llama2_7b_SDE | Mixtral_DE | 6.79X10-32 | TRUE |
| Llama2_13b_SDE | Mixtral_DE | 3.25X10-22 | TRUE |
| Llama3_8b_SDE | Mixtral_DE | 7.16X10-14 | TRUE |
| Mixtral_DE | Mistral_SDE | 4.28X10-01 | FALSE |
| Mixtral_SDE | Mixtral_DE | 2.42X10-02 | TRUE |
| Llama2_7b_CSDE | Mixtral_DE | 2.58X10-29 | TRUE |
| Mixtral_DE | Llama2_13b_CSDE | 6.81X10-21 | TRUE |
| Llama3_8b_CSDE | Mixtral_DE | 1.19X10-05 | TRUE |
| Mistral_CSDE | Mixtral_DE | 8.13X10-30 | TRUE |
| Mixtral_CSDE | Mixtral_DE | 2.70X10-08 | TRUE |
| MedCAT_Fine-tuning | Mixtral_DE | 8.59X10-04 | TRUE |
| Llama2_7b_CDE | Llama2_13b_CDE | 2.95X10-10 | TRUE |
| Llama2_7b_CDE | Llama3_8b_CDE | 7.83X10-04 | TRUE |
| Llama2_7b_CDE | Mistral_CDE | 5.42X10-13 | TRUE |
| Llama2_7b_CDE | Mixtral_CDE | 6.48X10-11 | TRUE |
| Llama2_7b_CDE | Llama2_7b_SDE | 2.61X10-01 | FALSE |
| Llama2_7b_CDE | Llama2_13b_SDE | 2.88X10-01 | FALSE |
| Llama2_7b_CDE | Llama3_8b_SDE | 2.27X10-03 | TRUE |
| Llama2_7b_CDE | Mistral_SDE | 9.13X10-29 | TRUE |
| Llama2_7b_CDE | Mixtral_SDE | 2.72X10-13 | TRUE |
| Llama2_7b_CDE | Llama2_7b_CSDE | 5.12X10-01 | FALSE |
| Llama2_7b_CDE | Llama2_13b_CSDE | 2.32X10-05 | TRUE |
| Llama2_7b_CDE | Llama3_8b_CSDE | 4.67X10-09 | TRUE |
| Llama2_7b_CDE | Mistral_CSDE | 3.83X10-01 | FALSE |
| Mixtral_CSDE | Llama2_7b_CDE | 1.94X10-07 | TRUE |
| Llama2_7b_CDE | MedCAT_Fine-tuning | 1.49X10-13 | TRUE |
| Llama3_8b_CDE | Llama2_13b_CDE | 4.20X10-02 | TRUE |
| Mistral_CDE | Llama2_13b_CDE | 8.49X10-02 | FALSE |
| Mixtral_CDE | Llama2_13b_CDE | 3.61X10-01 | FALSE |
| Llama2_7b_SDE | Llama2_13b_CDE | 1.64X10-20 | TRUE |
| Llama2_13b_CDE | Llama2_13b_SDE | 2.11X10-08 | TRUE |
| Llama3_8b_SDE | Llama2_13b_CDE | 7.49X10-08 | TRUE |
| Llama2_13b_CDE | Mistral_SDE | 4.46X10-07 | TRUE |
| Mixtral_SDE | Llama2_13b_CDE | 2.48X10-01 | FALSE |
| Llama2_7b_CSDE | Llama2_13b_CDE | 2.30X10-17 | TRUE |
| Llama2_13b_CDE | Llama2_13b_CSDE | 7.70X10-04 | TRUE |
| Llama2_13b_CDE | Llama3_8b_CSDE | 1.38X10-02 | TRUE |
| Mistral_CSDE | Llama2_13b_CDE | 2.80X10-16 | TRUE |
| Mixtral_CSDE | Llama2_13b_CDE | 2.27X10-06 | TRUE |
| Llama2_13b_CDE | MedCAT_Fine-tuning | 8.71X10-02 | FALSE |
| Mistral_CDE | Llama3_8b_CDE | 3.50X10-01 | FALSE |
| Llama3_8b_CDE | Mixtral_CDE | 1.92X10-05 | TRUE |
| Llama2_7b_SDE | Llama3_8b_CDE | 6.40X10-06 | TRUE |
| Llama3_8b_CDE | Llama2_13b_SDE | 6.78X10-08 | TRUE |
| Llama3_8b_SDE | Llama3_8b_CDE | 9.09X10-02 | FALSE |
| Llama3_8b_CDE | Mistral_SDE | 3.98X10-12 | TRUE |
| Llama3_8b_CDE | Mixtral_SDE | 3.02X10-07 | TRUE |
| Llama2_7b_CSDE | Llama3_8b_CDE | 2.96X10-10 | TRUE |
| Llama3_8b_CDE | Llama2_13b_CSDE | 4.33X10-02 | TRUE |
| Llama3_8b_CDE | Llama3_8b_CSDE | 3.98X10-01 | FALSE |
| Mistral_CSDE | Llama3_8b_CDE | 1.50X10-05 | TRUE |
| Mixtral_CSDE | Llama3_8b_CDE | 2.18X10-01 | FALSE |
| Llama3_8b_CDE | MedCAT_Fine-tuning | 2.01X10-01 | FALSE |
| Mistral_CDE | Mixtral_CDE | 2.83X10-06 | TRUE |
| Mistral_CDE | Llama2_7b_SDE | 3.22X10-09 | TRUE |
| Mistral_CDE | Llama2_13b_SDE | 4.59X10-06 | TRUE |
| Mistral_CDE | Llama3_8b_SDE | 3.84X10-02 | TRUE |
| Mistral_CDE | Mistral_SDE | 8.23X10-09 | TRUE |
| Mistral_CDE | Mixtral_SDE | 3.22X10-02 | TRUE |
| Mistral_CDE | Llama2_7b_CSDE | 6.56X10-12 | TRUE |
| Mistral_CDE | Llama2_13b_CSDE | 7.95X10-07 | TRUE |
| Mistral_CDE | Llama3_8b_CSDE | 8.13X10-02 | FALSE |
| Mistral_CDE | Mistral_CSDE | 9.10X10-06 | TRUE |
| Mixtral_CSDE | Mistral_CDE | 4.11X10-02 | TRUE |
| Mistral_CDE | MedCAT_Fine-tuning | 4.33X10-01 | FALSE |
| Llama2_7b_SDE | Mixtral_CDE | 7.57X10-14 | TRUE |
| Mixtral_CDE | Llama2_13b_SDE | 2.64X10-16 | TRUE |
| Llama3_8b_SDE | Mixtral_CDE | 2.05X10-08 | TRUE |
| Mixtral_CDE | Mistral_SDE | 9.40X10-06 | TRUE |
| Mixtral_SDE | Mixtral_CDE | 4.26X10-01 | FALSE |
| Llama2_7b_CSDE | Mixtral_CDE | 6.16X10-13 | TRUE |
| Mixtral_CDE | Llama2_13b_CSDE | 2.61X10-06 | TRUE |
| Mixtral_CDE | Llama3_8b_CSDE | 1.18X10-02 | TRUE |
| Mistral_CSDE | Mixtral_CDE | 1.04X10-14 | TRUE |
| Mixtral_CSDE | Mixtral_CDE | 3.70X10-02 | TRUE |
| Mixtral_CDE | MedCAT_Fine-tuning | 8.25X10-05 | TRUE |
| Llama2_7b_SDE | Llama2_13b_SDE | 8.87X10-02 | FALSE |
| Llama2_7b_SDE | Llama3_8b_SDE | 2.63X10-10 | TRUE |
| Llama2_7b_SDE | Mistral_SDE | 4.59X10-33 | TRUE |
| Llama2_7b_SDE | Mixtral_SDE | 1.78X10-17 | TRUE |
| Llama2_7b_SDE | Llama2_7b_CSDE | 3.09X10-01 | FALSE |
| Llama2_7b_SDE | Llama2_13b_CSDE | 7.00X10-03 | TRUE |
| Llama2_7b_SDE | Llama3_8b_CSDE | 9.55X10-10 | TRUE |
| Llama2_7b_SDE | Mistral_CSDE | 4.01X10-01 | FALSE |
| Mixtral_CSDE | Llama2_7b_SDE | 1.20X10-10 | TRUE |
| Llama2_7b_SDE | MedCAT_Fine-tuning | 7.02X10-11 | TRUE |
| Llama3_8b_SDE | Llama2_13b_SDE | 1.50X10-02 | TRUE |
| Llama2_13b_SDE | Mistral_SDE | 2.93X10-21 | TRUE |
| Mixtral_SDE | Llama2_13b_SDE | 6.86X10-12 | TRUE |
| Llama2_7b_CSDE | Llama2_13b_SDE | 2.10X10-01 | FALSE |
| Llama2_13b_SDE | Llama2_13b_CSDE | 1.08X10-01 | FALSE |
| Llama2_13b_SDE | Llama3_8b_CSDE | 4.57X10-02 | TRUE |
| Mistral_CSDE | Llama2_13b_SDE | 2.01X10-01 | FALSE |
| Mixtral_CSDE | Llama2_13b_SDE | 8.92X10-05 | TRUE |
| Llama2_13b_SDE | MedCAT_Fine-tuning | 4.62X10-11 | TRUE |
| Llama3_8b_SDE | Mistral_SDE | 8.99X10-07 | TRUE |
| Llama3_8b_SDE | Mixtral_SDE | 1.92X10-07 | TRUE |
| Llama2_7b_CSDE | Llama3_8b_SDE | 2.48X10-08 | TRUE |
| Llama3_8b_SDE | Llama2_13b_CSDE | 2.89X10-05 | TRUE |
| Llama3_8b_SDE | Llama3_8b_CSDE | 3.03X10-01 | FALSE |
| Llama3_8b_SDE | Mistral_CSDE | 9.22X10-09 | TRUE |
| Mixtral_CSDE | Llama3_8b_SDE | 3.84X10-01 | FALSE |
| Llama3_8b_SDE | MedCAT_Fine-tuning | 2.57X10-02 | TRUE |
| Mixtral_SDE | Mistral_SDE | 4.85X10-02 | TRUE |
| Llama2_7b_CSDE | Mistral_SDE | 2.90X10-22 | TRUE |
| Llama2_13b_CSDE | Mistral_SDE | 1.63X10-22 | TRUE |
| Llama3_8b_CSDE | Mistral_SDE | 2.95X10-07 | TRUE |
| Mistral_CSDE | Mistral_SDE | 6.22X10-29 | TRUE |
| Mixtral_CSDE | Mistral_SDE | 9.84X10-14 | TRUE |
| MedCAT_Fine-tuning | Mistral_SDE | 9.51X10-09 | TRUE |
| Llama2_7b_CSDE | Mixtral_SDE | 3.89X10-20 | TRUE |
| Mixtral_SDE | Llama2_13b_CSDE | 6.11X10-13 | TRUE |
| Mixtral_SDE | Llama3_8b_CSDE | 1.25X10-03 | TRUE |
| Mistral_CSDE | Mixtral_SDE | 5.61X10-14 | TRUE |
| Mixtral_CSDE | Mixtral_SDE | 9.61X10-07 | TRUE |
| Mixtral_SDE | MedCAT_Fine-tuning | 2.84X10-02 | TRUE |
| Llama2_7b_CSDE | Llama2_13b_CSDE | 1.61X10-02 | TRUE |
| Llama2_7b_CSDE | Llama3_8b_CSDE | 4.04X10-04 | TRUE |
| Llama2_7b_CSDE | Mistral_CSDE | 4.02X10-01 | FALSE |
| Mixtral_CSDE | Llama2_7b_CSDE | 3.85X10-09 | TRUE |
| Llama2_7b_CSDE | MedCAT_Fine-tuning | 2.10X10-07 | TRUE |
| Llama3_8b_CSDE | Llama2_13b_CSDE | 3.19X10-05 | TRUE |
| Mistral_CSDE | Llama2_13b_CSDE | 7.08X10-03 | TRUE |
| Mixtral_CSDE | Llama2_13b_CSDE | 7.06X10-06 | TRUE |
| MedCAT_Fine-tuning | Llama2_13b_CSDE | 8.44X10-08 | TRUE |
| Mistral_CSDE | Llama3_8b_CSDE | 3.13X10-02 | TRUE |
| Mixtral_CSDE | Llama3_8b_CSDE | 3.11X10-01 | FALSE |
| Llama3_8b_CSDE | MedCAT_Fine-tuning | 5.03X10-02 | FALSE |
| Mixtral_CSDE | Mistral_CSDE | 7.75X10-07 | TRUE |
| Mistral_CSDE | MedCAT_Fine-tuning | 7.75X10-16 | TRUE |
| Mixtral_CSDE | MedCAT_Fine-tuning | 1.12X10-02 | TRUE |

Corrected statistical comparison results of seizure type extraction

| **Model&Method 1** | **Model&Method 2** | **Corrected p-value** | **Significant(p<0.05)** |
| --- | --- | --- | --- |
| Epileptologists_Manually | Llama2_7b_DE | 8.25X10-14 | TRUE |
| Epileptologists_Manually | Llama2_13b_DE | 4.72X10-17 | TRUE |
| Epileptologists_Manually | Llama3_8b_DE | 7.79X10-47 | TRUE |
| Epileptologists_Manually | Mistral_DE | 3.83X10-43 | TRUE |
| Epileptologists_Manually | Mixtral_DE | 2.01X10-45 | TRUE |
| Epileptologists_Manually | Llama2_7b_CDE | 5.97X10-15 | TRUE |
| Epileptologists_Manually | Llama2_13b_CDE | 3.54X10-28 | TRUE |
| Epileptologists_Manually | Llama3_8b_CDE | 8.94X10-56 | TRUE |
| Epileptologists_Manually | Mistral_CDE | 9.31X10-52 | TRUE |
| Epileptologists_Manually | Mixtral_CDE | 2.01X10-54 | TRUE |
| Epileptologists_Manually | Llama2_7b_SDE | 6.56X10-13 | TRUE |
| Epileptologists_Manually | Llama2_13b_SDE | 2.72X10-05 | TRUE |
| Epileptologists_Manually | Llama3_8b_SDE | 7.42X10-43 | TRUE |
| Epileptologists_Manually | Mistral_SDE | 7.72X10-51 | TRUE |
| Epileptologists_Manually | Mixtral_SDE | 4.27X10-36 | TRUE |
| Epileptologists_Manually | Llama2_7b_CSDE | 2.61X10-15 | TRUE |
| Epileptologists_Manually | Llama2_13b_CSDE | 3.09X10-18 | TRUE |
| Epileptologists_Manually | Llama3_8b_CSDE | 9.71X10-38 | TRUE |
| Epileptologists_Manually | Mistral_CSDE | 3.70X10-16 | TRUE |
| Epileptologists_Manually | Mixtral_CSDE | 1.83X10-22 | TRUE |
| Epileptologists_Manually | MedCAT_Fine-tuning | 3.53X10-49 | TRUE |
| Llama2_13b_DE | Llama2_7b_DE | 7.08X10-03 | TRUE |
| Llama2_7b_DE | Llama3_8b_DE | 3.53X10-18 | TRUE |
| Llama2_7b_DE | Mistral_DE | 4.30X10-05 | TRUE |
| Llama2_7b_DE | Mixtral_DE | 1.18X10-17 | TRUE |
| Llama2_7b_DE | Llama2_7b_CDE | 7.47X10-02 | FALSE |
| Llama2_13b_CDE | Llama2_7b_DE | 1.44X10-12 | TRUE |
| Llama2_7b_DE | Llama3_8b_CDE | 5.89X10-15 | TRUE |
| Llama2_7b_DE | Mistral_CDE | 5.21X10-17 | TRUE |
| Llama2_7b_DE | Mixtral_CDE | 8.73X10-16 | TRUE |
| Llama2_7b_DE | Llama2_7b_SDE | 4.75X10-01 | FALSE |
| Llama2_7b_DE | Llama2_13b_SDE | 4.15X10-01 | FALSE |
| Llama2_7b_DE | Llama3_8b_SDE | 1.52X10-02 | TRUE |
| Llama2_7b_DE | Mistral_SDE | 2.87X10-19 | TRUE |
| Mixtral_SDE | Llama2_7b_DE | 6.34X10-04 | TRUE |
| Llama2_7b_DE | Llama2_7b_CSDE | 9.64X10-03 | TRUE |
| Llama2_7b_DE | Llama2_13b_CSDE | 4.15X10-01 | FALSE |
| Llama2_7b_DE | Llama3_8b_CSDE | 1.92X10-05 | TRUE |
| Mistral_CSDE | Llama2_7b_DE | 3.07X10-02 | TRUE |
| Mixtral_CSDE | Llama2_7b_DE | 6.25X10-12 | TRUE |
| Llama2_7b_DE | MedCAT_Fine-tuning | 3.74X10-14 | TRUE |
| Llama2_13b_DE | Llama3_8b_DE | 3.60X10-02 | TRUE |
| Llama2_13b_DE | Mistral_DE | 4.43X10-08 | TRUE |
| Llama2_13b_DE | Mixtral_DE | 7.70X10-12 | TRUE |
| Llama2_13b_DE | Llama2_7b_CDE | 3.82X10-03 | TRUE |
| Llama2_13b_DE | Llama2_13b_CDE | 8.52X10-03 | TRUE |
| Llama2_13b_DE | Llama3_8b_CDE | 8.93X10-11 | TRUE |
| Llama2_13b_DE | Mistral_CDE | 7.14X10-08 | TRUE |
| Llama2_13b_DE | Mixtral_CDE | 4.95X10-10 | TRUE |
| Llama2_13b_DE | Llama2_7b_SDE | 3.97X10-02 | TRUE |
| Llama2_13b_DE | Llama2_13b_SDE | 7.99X10-05 | TRUE |
| Llama2_13b_DE | Llama3_8b_SDE | 2.08X10-02 | TRUE |
| Llama2_13b_DE | Mistral_SDE | 5.78X10-14 | TRUE |
| Llama2_13b_DE | Mixtral_SDE | 2.93X10-05 | TRUE |
| Llama2_13b_DE | Llama2_7b_CSDE | 6.94X10-09 | TRUE |
| Llama2_13b_DE | Llama2_13b_CSDE | 4.02X10-10 | TRUE |
| Llama2_13b_DE | Llama3_8b_CSDE | 2.17X10-06 | TRUE |
| Llama2_13b_DE | Mistral_CSDE | 7.42X10-08 | TRUE |
| Llama2_13b_DE | Mixtral_CSDE | 4.86X10-04 | TRUE |
| Llama2_13b_DE | MedCAT_Fine-tuning | 4.73X10-07 | TRUE |
| Mistral_DE | Llama3_8b_DE | 6.01X10-01 | FALSE |
| Llama3_8b_DE | Mixtral_DE | 5.48X10-01 | FALSE |
| Llama2_7b_CDE | Llama3_8b_DE | 5.40X10-08 | TRUE |
| Llama2_13b_CDE | Llama3_8b_DE | 3.76X10-04 | TRUE |
| Llama3_8b_DE | Llama3_8b_CDE | 7.04X10-02 | FALSE |
| Mistral_CDE | Llama3_8b_DE | 9.60X10-02 | FALSE |
| Llama3_8b_DE | Mixtral_CDE | 3.07X10-01 | FALSE |
| Llama3_8b_DE | Llama2_7b_SDE | 7.63X10-10 | TRUE |
| Llama3_8b_DE | Llama2_13b_SDE | 3.08X10-17 | TRUE |
| Llama3_8b_SDE | Llama3_8b_DE | 3.56X10-01 | FALSE |
| Llama3_8b_DE | Mistral_SDE | 3.63X10-02 | TRUE |
| Mixtral_SDE | Llama3_8b_DE | 1.88X10-02 | TRUE |
| Llama3_8b_DE | Llama2_7b_CSDE | 7.68X10-14 | TRUE |
| Llama3_8b_DE | Llama2_13b_CSDE | 5.21X10-12 | TRUE |
| Llama3_8b_DE | Llama3_8b_CSDE | 6.07X10-01 | FALSE |
| Mistral_CSDE | Llama3_8b_DE | 6.09X10-06 | TRUE |
| Mixtral_CSDE | Llama3_8b_DE | 5.33X10-04 | TRUE |
| Llama3_8b_DE | MedCAT_Fine-tuning | 6.23X10-01 | FALSE |
| Mistral_DE | Mixtral_DE | 4.53X10-01 | FALSE |
| Mistral_DE | Llama2_7b_CDE | 8.30X10-03 | TRUE |
| Llama2_13b_CDE | Mistral_DE | 9.51X10-04 | TRUE |
| Mistral_DE | Llama3_8b_CDE | 4.43X10-09 | TRUE |
| Mistral_DE | Mistral_CDE | 3.23X10-01 | FALSE |
| Mistral_DE | Mixtral_CDE | 3.71X10-01 | FALSE |
| Mistral_DE | Llama2_7b_SDE | 9.78X10-12 | TRUE |
| Mistral_DE | Llama2_13b_SDE | 4.43X10-15 | TRUE |
| Mistral_DE | Llama3_8b_SDE | 3.09X10-01 | FALSE |
| Mistral_DE | Mistral_SDE | 1.70X10-09 | TRUE |
| Mixtral_SDE | Mistral_DE | 7.91X10-08 | TRUE |
| Mistral_DE | Llama2_7b_CSDE | 5.94X10-14 | TRUE |
| Mistral_DE | Llama2_13b_CSDE | 2.07X10-04 | TRUE |
| Mistral_DE | Llama3_8b_CSDE | 6.78X10-01 | FALSE |
| Mistral_CSDE | Mistral_DE | 6.10X10-08 | TRUE |
| Mixtral_CSDE | Mistral_DE | 4.28X10-03 | TRUE |
| Mistral_DE | MedCAT_Fine-tuning | 5.31X10-01 | FALSE |
| Llama2_7b_CDE | Mixtral_DE | 6.86X10-07 | TRUE |
| Llama2_13b_CDE | Mixtral_DE | 5.28X10-04 | TRUE |
| Mixtral_DE | Llama3_8b_CDE | 5.13X10-03 | TRUE |
| Mistral_CDE | Mixtral_DE | 7.77X10-08 | TRUE |
| Mixtral_DE | Mixtral_CDE | 7.60X10-02 | FALSE |
| Mixtral_DE | Llama2_7b_SDE | 1.89X10-10 | TRUE |
| Mixtral_DE | Llama2_13b_SDE | 4.33X10-09 | TRUE |
| Llama3_8b_SDE | Mixtral_DE | 4.75X10-01 | FALSE |
| Mixtral_DE | Mistral_SDE | 1.83X10-04 | TRUE |
| Mixtral_SDE | Mixtral_DE | 7.64X10-09 | TRUE |
| Mixtral_DE | Llama2_7b_CSDE | 2.97X10-06 | TRUE |
| Mixtral_DE | Llama2_13b_CSDE | 3.39X10-10 | TRUE |
| Mixtral_DE | Llama3_8b_CSDE | 4.60X10-01 | FALSE |
| Mistral_CSDE | Mixtral_DE | 9.08X10-06 | TRUE |
| Mixtral_CSDE | Mixtral_DE | 9.80X10-07 | TRUE |
| Mixtral_DE | MedCAT_Fine-tuning | 6.04X10-01 | FALSE |
| Llama2_13b_CDE | Llama2_7b_CDE | 5.70X10-09 | TRUE |
| Llama2_7b_CDE | Llama3_8b_CDE | 1.44X10-19 | TRUE |
| Mistral_CDE | Llama2_7b_CDE | 1.45X10-12 | TRUE |
| Llama2_7b_CDE | Mixtral_CDE | 6.80X10-05 | TRUE |
| Llama2_7b_CDE | Llama2_7b_SDE | 3.46X10-01 | FALSE |
| Llama2_7b_CDE | Llama2_13b_SDE | 3.71X10-01 | FALSE |
| Llama2_7b_CDE | Llama3_8b_SDE | 2.97X10-02 | TRUE |
| Llama2_7b_CDE | Mistral_SDE | 5.36X10-14 | TRUE |
| Mixtral_SDE | Llama2_7b_CDE | 3.54X10-03 | TRUE |
| Llama2_7b_CDE | Llama2_7b_CSDE | 6.23X10-01 | FALSE |
| Llama2_7b_CDE | Llama2_13b_CSDE | 3.71X10-01 | FALSE |
| Llama2_7b_CDE | Llama3_8b_CSDE | 1.72X10-14 | TRUE |
| Mistral_CSDE | Llama2_7b_CDE | 5.10X10-09 | TRUE |
| Mixtral_CSDE | Llama2_7b_CDE | 8.07X10-09 | TRUE |
| Llama2_7b_CDE | MedCAT_Fine-tuning | 2.01X10-03 | TRUE |
| Llama2_13b_CDE | Llama3_8b_CDE | 6.69X10-06 | TRUE |
| Llama2_13b_CDE | Mistral_CDE | 4.52X10-02 | TRUE |
| Llama2_13b_CDE | Mixtral_CDE | 8.59X10-10 | TRUE |
| Llama2_13b_CDE | Llama2_7b_SDE | 5.74X10-11 | TRUE |
| Llama2_13b_CDE | Llama2_13b_SDE | 5.74X10-06 | TRUE |
| Llama2_13b_CDE | Llama3_8b_SDE | 4.45X10-02 | TRUE |
| Llama2_13b_CDE | Mistral_SDE | 1.04X10-02 | TRUE |
| Mixtral_SDE | Llama2_13b_CDE | 4.80X10-02 | TRUE |
| Llama2_13b_CDE | Llama2_7b_CSDE | 4.74X10-02 | TRUE |
| Llama2_13b_CDE | Llama2_13b_CSDE | 9.50X10-06 | TRUE |
| Llama2_13b_CDE | Llama3_8b_CSDE | 6.47X10-06 | TRUE |
| Llama2_13b_CDE | Mistral_CSDE | 2.45X10-02 | TRUE |
| Mixtral_CSDE | Llama2_13b_CDE | 4.18X10-04 | TRUE |
| Llama2_13b_CDE | MedCAT_Fine-tuning | 6.92X10-06 | TRUE |
| Mistral_CDE | Llama3_8b_CDE | 6.35X10-01 | FALSE |
| Llama3_8b_CDE | Mixtral_CDE | 5.42X10-01 | FALSE |
| Llama2_7b_SDE | Llama3_8b_CDE | 1.60X10-11 | TRUE |
| Llama2_13b_SDE | Llama3_8b_CDE | 3.25X10-14 | TRUE |
| Llama3_8b_SDE | Llama3_8b_CDE | 2.80X10-02 | TRUE |
| Mistral_SDE | Llama3_8b_CDE | 3.52X10-01 | FALSE |
| Mixtral_SDE | Llama3_8b_CDE | 4.10X10-08 | TRUE |
| Llama2_7b_CSDE | Llama3_8b_CDE | 3.35X10-19 | TRUE |
| Llama2_13b_CSDE | Llama3_8b_CDE | 3.21X10-14 | TRUE |
| Llama3_8b_CSDE | Llama3_8b_CDE | 3.09X10-01 | FALSE |
| Mistral_CSDE | Llama3_8b_CDE | 3.34X10-10 | TRUE |
| Mixtral_CSDE | Llama3_8b_CDE | 6.88X10-05 | TRUE |
| MedCAT_Fine-tuning | Llama3_8b_CDE | 6.89X10-06 | TRUE |
| Mistral_CDE | Mixtral_CDE | 6.07X10-01 | FALSE |
| Mistral_CDE | Llama2_7b_SDE | 1.50X10-13 | TRUE |
| Mistral_CDE | Llama2_13b_SDE | 5.33X10-18 | TRUE |
| Mistral_CDE | Llama3_8b_SDE | 6.42X10-03 | TRUE |
| Mistral_CDE | Mistral_SDE | 3.40X10-01 | FALSE |
| Mixtral_SDE | Mistral_CDE | 4.29X10-02 | TRUE |
| Mistral_CDE | Llama2_7b_CSDE | 7.69X10-15 | TRUE |
| Mistral_CDE | Llama2_13b_CSDE | 5.34X10-08 | TRUE |
| Mistral_CDE | Llama3_8b_CSDE | 3.13X10-01 | FALSE |
| Mistral_CSDE | Mistral_CDE | 9.15X10-04 | TRUE |
| Mixtral_CSDE | Mistral_CDE | 1.90X10-05 | TRUE |
| Mistral_CDE | MedCAT_Fine-tuning | 7.12X10-02 | FALSE |
| Llama2_7b_SDE | Mixtral_CDE | 9.68X10-19 | TRUE |
| Llama2_13b_SDE | Mixtral_CDE | 4.97X10-20 | TRUE |
| Llama3_8b_SDE | Mixtral_CDE | 3.59X10-02 | TRUE |
| Mistral_SDE | Mixtral_CDE | 5.65X10-02 | FALSE |
| Mixtral_SDE | Mixtral_CDE | 8.98X10-04 | TRUE |
| Llama2_7b_CSDE | Mixtral_CDE | 4.68X10-08 | TRUE |
| Llama2_13b_CSDE | Mixtral_CDE | 5.42X10-13 | TRUE |
| Llama3_8b_CSDE | Mixtral_CDE | 3.60X10-01 | FALSE |
| Mistral_CSDE | Mixtral_CDE | 1.76X10-04 | TRUE |
| Mixtral_CSDE | Mixtral_CDE | 2.68X10-03 | TRUE |
| MedCAT_Fine-tuning | Mixtral_CDE | 9.64X10-02 | FALSE |
| Llama2_13b_SDE | Llama2_7b_SDE | 6.20X10-01 | FALSE |
| Llama3_8b_SDE | Llama2_7b_SDE | 1.34X10-02 | TRUE |
| Llama2_7b_SDE | Mistral_SDE | 6.93X10-17 | TRUE |
| Mixtral_SDE | Llama2_7b_SDE | 4.30X10-12 | TRUE |
| Llama2_7b_CSDE | Llama2_7b_SDE | 3.13X10-01 | FALSE |
| Llama2_7b_SDE | Llama2_13b_CSDE | 6.20X10-01 | FALSE |
| Llama3_8b_CSDE | Llama2_7b_SDE | 6.92X10-05 | TRUE |
| Mistral_CSDE | Llama2_7b_SDE | 6.52X10-03 | TRUE |
| Mixtral_CSDE | Llama2_7b_SDE | 7.33X10-05 | TRUE |
| Llama2_7b_SDE | MedCAT_Fine-tuning | 8.29X10-06 | TRUE |
| Llama3_8b_SDE | Llama2_13b_SDE | 4.71X10-10 | TRUE |
| Llama2_13b_SDE | Mistral_SDE | 1.72X10-16 | TRUE |
| Mixtral_SDE | Llama2_13b_SDE | 4.93X10-02 | TRUE |
| Llama2_7b_CSDE | Llama2_13b_SDE | 3.56X10-01 | FALSE |
| Llama2_13b_SDE | Llama2_13b_CSDE | 7.06X10-01 | FALSE |
| Llama2_13b_SDE | Llama3_8b_CSDE | 6.45X10-16 | TRUE |
| Mistral_CSDE | Llama2_13b_SDE | 4.93X10-02 | TRUE |
| Mixtral_CSDE | Llama2_13b_SDE | 6.85X10-10 | TRUE |
| Llama2_13b_SDE | MedCAT_Fine-tuning | 8.96X10-05 | TRUE |
| Llama3_8b_SDE | Mistral_SDE | 3.38X10-05 | TRUE |
| Mixtral_SDE | Llama3_8b_SDE | 1.64X10-08 | TRUE |
| Llama3_8b_SDE | Llama2_7b_CSDE | 1.92X10-07 | TRUE |
| Llama3_8b_SDE | Llama2_13b_CSDE | 1.40X10-08 | TRUE |
| Llama3_8b_SDE | Llama3_8b_CSDE | 3.02X10-01 | FALSE |
| Mistral_CSDE | Llama3_8b_SDE | 6.73X10-11 | TRUE |
| Mixtral_CSDE | Llama3_8b_SDE | 4.52X10-02 | TRUE |
| Llama3_8b_SDE | MedCAT_Fine-tuning | 3.73X10-01 | FALSE |
| Mixtral_SDE | Mistral_SDE | 1.97X10-02 | TRUE |
| Llama2_7b_CSDE | Mistral_SDE | 8.23X10-07 | TRUE |
| Llama2_13b_CSDE | Mistral_SDE | 8.98X10-22 | TRUE |
| Llama3_8b_CSDE | Mistral_SDE | 8.22X10-06 | TRUE |
| Mistral_CSDE | Mistral_SDE | 4.42X10-13 | TRUE |
| Mixtral_CSDE | Mistral_SDE | 5.16X10-03 | TRUE |
| MedCAT_Fine-tuning | Mistral_SDE | 2.66X10-02 | TRUE |
| Mixtral_SDE | Llama2_7b_CSDE | 1.04X10-03 | TRUE |
| Mixtral_SDE | Llama2_13b_CSDE | 6.91X10-07 | TRUE |
| Mixtral_SDE | Llama3_8b_CSDE | 4.97X10-09 | TRUE |
| Mixtral_SDE | Mistral_CSDE | 6.63X10-05 | TRUE |
| Mixtral_CSDE | Mixtral_SDE | 4.08X10-02 | TRUE |
| Mixtral_SDE | MedCAT_Fine-tuning | 4.49X10-04 | TRUE |
| Llama2_7b_CSDE | Llama2_13b_CSDE | 3.56X10-01 | FALSE |
| Llama2_7b_CSDE | Llama3_8b_CSDE | 9.48X10-07 | TRUE |
| Mistral_CSDE | Llama2_7b_CSDE | 6.65X10-06 | TRUE |
| Mixtral_CSDE | Llama2_7b_CSDE | 2.83X10-02 | TRUE |
| Llama2_7b_CSDE | MedCAT_Fine-tuning | 3.82X10-07 | TRUE |
| Llama3_8b_CSDE | Llama2_13b_CSDE | 6.29X10-05 | TRUE |
| Mistral_CSDE | Llama2_13b_CSDE | 3.95X10-06 | TRUE |
| Mixtral_CSDE | Llama2_13b_CSDE | 1.58X10-08 | TRUE |
| Llama2_13b_CSDE | MedCAT_Fine-tuning | 3.75X10-10 | TRUE |
| Mistral_CSDE | Llama3_8b_CSDE | 4.29X10-03 | TRUE |
| Mixtral_CSDE | Llama3_8b_CSDE | 3.82X10-08 | TRUE |
| Llama3_8b_CSDE | MedCAT_Fine-tuning | 5.48X10-01 | FALSE |
| Mixtral_CSDE | Mistral_CSDE | 5.44X10-06 | TRUE |
| Mistral_CSDE | MedCAT_Fine-tuning | 4.86X10-14 | TRUE |
| Mixtral_CSDE | MedCAT_Fine-tuning | 9.28X10-10 | TRUE |

Corrected statistical comparison results of current ASM extraction

| **Model&Method 1** | **Model&Method 2** | **Corrected p-value** | **Significant(p<0.05)** |
| --- | --- | --- | --- |
| Epileptologists_Manually | Llama2_7b_DE | 7.94X10-22 | TRUE |
| Epileptologists_Manually | Llama2_13b_DE | 1.76X10-06 | TRUE |
| Epileptologists_Manually | Llama3_8b_DE | 7.85X10-13 | TRUE |
| Epileptologists_Manually | Mistral_DE | 8.89X10-14 | TRUE |
| Epileptologists_Manually | Mixtral_DE | 2.52X10-09 | TRUE |
| Epileptologists_Manually | Llama2_7b_SDE | 1.33X10-08 | TRUE |
| Epileptologists_Manually | Llama2_13b_SDE | 2.32X10-43 | TRUE |
| Epileptologists_Manually | Llama3_8b_SDE | 2.09X10-02 | TRUE |
| Epileptologists_Manually | Mistral_SDE | 1.69X10-15 | TRUE |
| Epileptologists_Manually | Mixtral_SDE | 4.33X10-02 | TRUE |
| Epileptologists_Manually | MedCAT_Fine-tuning | 4.41X10-12 | TRUE |
| Llama2_13b_DE | Llama2_7b_DE | 5.31X10-09 | TRUE |
| Llama2_7b_DE | Llama3_8b_DE | 4.16X10-04 | TRUE |
| Mistral_DE | Llama2_7b_DE | 6.00X10-01 | FALSE |
| Mixtral_DE | Llama2_7b_DE | 7.18X10-02 | FALSE |
| Llama2_7b_SDE | Llama2_7b_DE | 3.23X10-02 | TRUE |
| Llama2_7b_DE | Llama2_13b_SDE | 9.05X10-13 | TRUE |
| Llama3_8b_SDE | Llama2_7b_DE | 6.21X10-05 | TRUE |
| Llama2_7b_DE | Mistral_SDE | 3.94X10-02 | TRUE |
| Mixtral_SDE | Llama2_7b_DE | 7.19X10-11 | TRUE |
| Llama2_7b_DE | MedCAT_Fine-tuning | 5.13X10-04 | TRUE |
| Llama2_13b_DE | Llama3_8b_DE | 2.35X10-02 | TRUE |
| Llama2_13b_DE | Mistral_DE | 1.46X10-02 | TRUE |
| Llama2_13b_DE | Mixtral_DE | 7.90X10-12 | TRUE |
| Llama2_13b_DE | Llama2_7b_SDE | 2.61X10-04 | TRUE |
| Llama2_13b_DE | Llama2_13b_SDE | 5.01X10-39 | TRUE |
| Llama2_13b_DE | Llama3_8b_SDE | 4.73X10-06 | TRUE |
| Llama2_13b_DE | Mistral_SDE | 9.34X10-18 | TRUE |
| Mixtral_SDE | Llama2_13b_DE | 3.11X10-01 | FALSE |
| Llama2_13b_DE | MedCAT_Fine-tuning | 3.14X10-02 | TRUE |
| Mistral_DE | Llama3_8b_DE | 3.97X10-04 | TRUE |
| Mixtral_DE | Llama3_8b_DE | 4.17X10-01 | FALSE |
| Llama2_7b_SDE | Llama3_8b_DE | 4.11X10-11 | TRUE |
| Llama3_8b_DE | Llama2_13b_SDE | 1.74X10-18 | TRUE |
| Llama3_8b_SDE | Llama3_8b_DE | 4.55X10-10 | TRUE |
| Llama3_8b_DE | Mistral_SDE | 5.73X10-02 | FALSE |
| Mixtral_SDE | Llama3_8b_DE | 3.76X10-08 | TRUE |
| Llama3_8b_DE | MedCAT_Fine-tuning | 4.11X10-01 | FALSE |
| Mistral_DE | Mixtral_DE | 3.73X10-01 | FALSE |
| Llama2_7b_SDE | Mistral_DE | 3.59X10-02 | TRUE |
| Mistral_DE | Llama2_13b_SDE | 8.47X10-15 | TRUE |
| Llama3_8b_SDE | Mistral_DE | 5.27X10-02 | FALSE |
| Mistral_DE | Mistral_SDE | 9.34X10-05 | TRUE |
| Mixtral_SDE | Mistral_DE | 3.73X10-06 | TRUE |
| Mistral_DE | MedCAT_Fine-tuning | 9.41X10-07 | TRUE |
| Llama2_7b_SDE | Mixtral_DE | 2.53X10-03 | TRUE |
| Mixtral_DE | Llama2_13b_SDE | 8.58X10-13 | TRUE |
| Llama3_8b_SDE | Mixtral_DE | 3.27X10-02 | TRUE |
| Mixtral_DE | Mistral_SDE | 2.13X10-02 | TRUE |
| Mixtral_SDE | Mixtral_DE | 3.99X10-13 | TRUE |
| Mixtral_DE | MedCAT_Fine-tuning | 8.68X10-02 | FALSE |
| Llama2_7b_SDE | Llama2_13b_SDE | 8.89X10-26 | TRUE |
| Llama3_8b_SDE | Llama2_7b_SDE | 4.77X10-01 | FALSE |
| Llama2_7b_SDE | Mistral_SDE | 3.94X10-14 | TRUE |
| Mixtral_SDE | Llama2_7b_SDE | 1.73X10-07 | TRUE |
| Llama2_7b_SDE | MedCAT_Fine-tuning | 8.56X10-05 | TRUE |
| Llama3_8b_SDE | Llama2_13b_SDE | 6.30X10-29 | TRUE |
| Mistral_SDE | Llama2_13b_SDE | 7.88X10-07 | TRUE |
| Mixtral_SDE | Llama2_13b_SDE | 7.34X10-34 | TRUE |
| MedCAT_Fine-tuning | Llama2_13b_SDE | 4.41X10-02 | TRUE |
| Llama3_8b_SDE | Mistral_SDE | 1.76X10-12 | TRUE |
| Mixtral_SDE | Llama3_8b_SDE | 3.18X10-02 | TRUE |
| Llama3_8b_SDE | MedCAT_Fine-tuning | 1.47X10-02 | TRUE |
| Mixtral_SDE | Mistral_SDE | 8.77X10-06 | TRUE |
| MedCAT_Fine-tuning | Mistral_SDE | 4.64X10-01 | FALSE |
| Mixtral_SDE | MedCAT_Fine-tuning | 8.11X10-15 | TRUE |

Corrected statistical comparison results of associated symptom extraction

| **Model&Method 1** | **Model&Method 2** | **Corrected p-value** | **Significant(p<0.05)** |
| --- | --- | --- | --- |
| Epileptologists_Manually | Llama2_7b_DE | 1.79X10-02 | TRUE |
| Epileptologists_Manually | Llama2_13b_DE | 4.36X10-30 | TRUE |
| Epileptologists_Manually | Llama3_8b_DE | 3.83X10-02 | TRUE |
| Epileptologists_Manually | Mistral_DE | 4.14X10-04 | TRUE |
| Epileptologists_Manually | Mixtral_DE | 2.79X10-07 | TRUE |
| Epileptologists_Manually | Llama2_7b_SDE | 2.20X10-05 | TRUE |
| Epileptologists_Manually | Llama2_13b_SDE | 4.20X10-36 | TRUE |
| Epileptologists_Manually | Llama3_8b_SDE | 4.78X10-06 | TRUE |
| Epileptologists_Manually | Mistral_SDE | 8.13X10-04 | TRUE |
| Epileptologists_Manually | Mixtral_SDE | 6.32X10-05 | TRUE |
| Epileptologists_Manually | MedCAT_Fine-tuning | 7.32X10-04 | TRUE |
| Llama2_7b_DE | Llama2_13b_DE | 1.22X10-21 | TRUE |
| Llama2_7b_DE | Llama3_8b_DE | 8.61X10-03 | TRUE |
| Mistral_DE | Llama2_7b_DE | 1.31X10-02 | TRUE |
| Llama2_7b_DE | Mixtral_DE | 9.42X10-07 | TRUE |
| Llama2_7b_DE | Llama2_7b_SDE | 7.37X10-02 | FALSE |
| Llama2_7b_DE | Llama2_13b_SDE | 7.99X10-23 | TRUE |
| Llama2_7b_DE | Llama3_8b_SDE | 4.85X10-04 | TRUE |
| Llama2_7b_DE | Mistral_SDE | 5.43X10-01 | FALSE |
| Llama2_7b_DE | Mixtral_SDE | 6.04X10-01 | FALSE |
| Llama2_7b_DE | MedCAT_Fine-tuning | 5.00X10-01 | FALSE |
| Llama3_8b_DE | Llama2_13b_DE | 4.21X10-12 | TRUE |
| Mistral_DE | Llama2_13b_DE | 2.32X10-29 | TRUE |
| Mixtral_DE | Llama2_13b_DE | 7.41X10-13 | TRUE |
| Llama2_7b_SDE | Llama2_13b_DE | 4.93X10-11 | TRUE |
| Llama2_13b_DE | Llama2_13b_SDE | 6.79X10-01 | FALSE |
| Llama3_8b_SDE | Llama2_13b_DE | 3.68X10-12 | TRUE |
| Mistral_SDE | Llama2_13b_DE | 4.72X10-23 | TRUE |
| Mixtral_SDE | Llama2_13b_DE | 9.78X10-17 | TRUE |
| MedCAT_Fine-tuning | Llama2_13b_DE | 1.49X10-22 | TRUE |
| Mistral_DE | Llama3_8b_DE | 2.00X10-05 | TRUE |
| Mixtral_DE | Llama3_8b_DE | 6.04X10-01 | FALSE |
| Llama3_8b_DE | Llama2_7b_SDE | 6.04X10-01 | FALSE |
| Llama3_8b_DE | Llama2_13b_SDE | 7.27X10-15 | TRUE |
| Llama3_8b_DE | Llama3_8b_SDE | 6.57X10-01 | FALSE |
| Mistral_SDE | Llama3_8b_DE | 1.63X10-02 | TRUE |
| Mixtral_SDE | Llama3_8b_DE | 4.79X10-06 | TRUE |
| MedCAT_Fine-tuning | Llama3_8b_DE | 7.08X10-02 | FALSE |
| Mistral_DE | Mixtral_DE | 2.47X10-08 | TRUE |
| Mistral_DE | Llama2_7b_SDE | 1.74X10-03 | TRUE |
| Mistral_DE | Llama2_13b_SDE | 5.91X10-24 | TRUE |
| Mistral_DE | Llama3_8b_SDE | 6.36X10-05 | TRUE |
| Mistral_DE | Mistral_SDE | 9.36X10-07 | TRUE |
| Mistral_DE | Mixtral_SDE | 3.02X10-06 | TRUE |
| Mistral_DE | MedCAT_Fine-tuning | 1.56X10-02 | TRUE |
| Mixtral_DE | Llama2_7b_SDE | 6.79X10-01 | FALSE |
| Mixtral_DE | Llama2_13b_SDE | 6.24X10-25 | TRUE |
| Mixtral_DE | Llama3_8b_SDE | 6.31X10-01 | FALSE |
| Mistral_SDE | Mixtral_DE | 5.38X10-09 | TRUE |
| Mixtral_SDE | Mixtral_DE | 4.58X10-09 | TRUE |
| Mixtral_DE | MedCAT_Fine-tuning | 8.08X10-02 | FALSE |
| Llama2_7b_SDE | Llama2_13b_SDE | 8.47X10-16 | TRUE |
| Llama3_8b_SDE | Llama2_7b_SDE | 6.31X10-01 | FALSE |
| Mistral_SDE | Llama2_7b_SDE | 3.34X10-07 | TRUE |
| Mixtral_SDE | Llama2_7b_SDE | 2.46X10-02 | TRUE |
| MedCAT_Fine-tuning | Llama2_7b_SDE | 6.97X10-02 | FALSE |
| Llama3_8b_SDE | Llama2_13b_SDE | 6.05X10-18 | TRUE |
| Mistral_SDE | Llama2_13b_SDE | 4.92X10-18 | TRUE |
| Mixtral_SDE | Llama2_13b_SDE | 1.71X10-24 | TRUE |
| MedCAT_Fine-tuning | Llama2_13b_SDE | 1.23X10-23 | TRUE |
| Mistral_SDE | Llama3_8b_SDE | 1.48X10-08 | TRUE |
| Mixtral_SDE | Llama3_8b_SDE | 6.44X10-09 | TRUE |
| MedCAT_Fine-tuning | Llama3_8b_SDE | 2.09X10-02 | TRUE |
| Mixtral_SDE | Mistral_SDE | 4.36X10-01 | FALSE |
| Mistral_SDE | MedCAT_Fine-tuning | 6.44X10-01 | FALSE |
| Mixtral_SDE | MedCAT_Fine-tuning | 4.07X10-01 | FALSE |
